# Facilitating genomic breeding in citrus via reference panel-based whole-genome imputation of low-coverage sequencing-derived genotype data

**DOI:** 10.1270/jsbbs.25061

**Published:** 2026-02-18

**Authors:** Tokurou Shimizu, Keisuke Nonaka

**Affiliations:** 1 Institute of Fruit Tree Science, NARO, 485-6 Okitsu Nakacho, Shimizu, Shizuoka 424-0292, Japan; 2 Laboratory of Plant DNA Analysis, Kazusa DNA Research Institute, 2-6-7 Kazusa-Kamatari, Kisarazu, Chiba 292-0818, Japan

**Keywords:** *Citrus*, genome, genomic-assisted breeding, whole-genome imputation, genomic prediction, genome-wide association studies, ancestry informative markers

## Abstract

The National Agriculture and Food Research Organization (NARO) is advancing “Citrus Breeding 2.0” to produce diverse, high-quality hybrid citrus cultivars more efficiently by integrating genomic prediction, genome-wide association studies (GWAS), and pedigree data. Reduced representation sequencing (RRS) methods, such as RAD-Seq, ddRAD-Seq, and GRAS-Di, facilitate large-scale, cost-effective genotyping; however, variable loci hinder cross-platform comparisons, limiting model reuse and GWAS follow-up. Therefore, we developed an Augmented Estimation of Unified Genotype (AEUG) workflow that converts RRS-derived genotypes into a unified set of predefined loci using a whole-genome resequencing reference panel that shares a common haplotype with target populations. Although Beagle-based whole-genome imputation achieved only 61.3–83.6% accuracy, genomic prediction for 17 fruit traits remained virtually unchanged after conversion, demonstrating the robustness of the workflow. The alignment of loci with ancestry informative markers for four pure citrus species also enabled the estimation of the ancestral origin of the trait-associated genomic regions. The AEUG workflow facilitates the integration and reuse of heterogeneous genotype datasets, enhances prediction accuracy, and enables ancestry-informed GWAS interpretation to accelerate citrus genomic breeding.

## Introduction

### Challenges and opportunities in citrus breeding at the National Agriculture and Food Research Organization (NARO)

Citrus species exhibit high genetic heterozygosity, which results in the segregation of diverse traits even within a single generation (Caruso *et al.* 2020, Cuenca *et al.* 2018, Hasegawa 2013, Raveh *et al.* 2020). Using this characteristic, the National Agriculture and Food Research Organization (NARO) developed 39 hybrid cultivars by selecting superior seedlings from genetically diverse parental crosses (Shimizu 2022).

Traditionally, the NARO Citrus breeding program has focused on hybrid cultivars in the domestic market (Hasegawa 2013). However, recently, the demand for new hybrids competing in global markets has rapidly increased (Shimizu 2022, Shimizu and Nonaka 2025). In response, the current program is designed to develop high-quality hybrid cultivars with unique fruit characteristics tailored for diverse international markets while simultaneously reducing the breeding cycle (Shimizu 2022).

A considerable challenge is the long juvenile period, at least 7 years from sowing to first flowering, combined with the substantial resources required for phenotypic evaluation, which notably extend the breeding cycle (Shimizu and Nonaka 2025). For example, hybrid scions bred by NARO require an average of 20.4 years to be released (Shimizu 2022). Thus, accelerating the development of new scion cultivars with both high quality and originality requires increasing the proportion of promising seedlings within a single generation rather than relying on gradual improvement through repeated crossing.

However, the large size of fruit trees and the high cost of maintaining seedlings in the field limit the scale of breeding efforts (Shimizu 2022).

### Efforts of citrus breeding 2.0

At NARO, 5,000–10,000 seeds are obtained annually from numerous hybrid combinations. They are germinated in greenhouses, grown for approximately 1 year, and then grafted onto trifoliate orange (*Poncirus* sp.; Karatachi) rootstocks in the field to reduce the time to first flowering. However, owing to limited field space, labor, and costs, only approximately 1,000 seedlings can be grafted yearly, and most seeds are discarded without trait evaluation. To address this limitation, predicting traits at the seedling stage and grafting potential individuals only facilitates all the germinated seedlings to be assessed for target traits (Shimizu 2020a, Shimizu *et al.* 2020b). This strategy increases the proportion of potential seedlings without expanding the field size.

The NARO hybrid breeding program evaluates over 20 traits, including fruit weight, sugar or soluble solid content, acidity, ease of peel, and rind and flesh firmness and color. Many of these traits are polygenic; therefore, the current MAS approaches, which rely on one or a few DNA markers, are limited and frequently demonstrate reduced effectiveness (Shimizu *et al.* 2020, Shimizu and Iwata 2024). Genomic prediction using genome-wide marker data is effective for citrus traits (Minamikawa *et al.* 2017), and genome-wide association studies (GWAS) have identified genomic regions associated with traits such as firmness, color, and ease of peeling.

In fruit trees, a long juvenile period hinders the development of test cross-populations for each trait (Badenes and Byrne 2012, Iwata *et al.* 2016, Luby and Shaw 2001). Therefore, the GWAS-based identification of trait-associated regions provides a valuable path for the development of DNA markers for MAS. Based on these achievements, NARO launched “Citrus Breeding 2.0”, an initiative that addresses three competing goals: expanding genetic diversity, improving fruit quality, and shortening breeding cycles (Shimizu 2022). This genomics-assisted breeding (GAB) approach (Iwata *et al.* 2016) integrates genome-wide genotype data with pedigree information, and its success depends on obtaining large-scale, high-quality genotyping data at a low cost.

### Use of low-cost genotyping methods for GAB

To meet the requirements of MAS, various genome-wide genotyping methods have been developed for plant breeding. Single-nucleotide polymorphism (SNP) markers are commonly used in GAB owing to their abundance and ease of handling (Batley 2015, Henry 2008, Iwata *et al.* 2016, Shimizu *et al.* 2020, Shimizu and Iwata 2024). These methods can be classified broadly into three categories.

1. **Microarray- or bead array-based genotyping** has high reproducibility, throughput, and low per-SNP costs, with access to extensive genetic information. Several large-scale citrus arrays have been developed (Fujii *et al.* 2012, Hiraoka *et al.* 2024, Minamikawa *et al.* 2017). However, these methods require extensive prior information on variant loci and provide limited flexibility in modifying target loci, making them less suitable for genetically diverse populations.

2. **Multiplex amplicon or targeted sequencing** uses multiplex PCR to amplify numerous genomic regions, followed by Next-Generation Sequencing (NGS) for variant detection (Nagano *et al.* 2020, Nishio *et al.* 2023, Onda *et al.* 2018). It provides high-precision genotyping for defined regions, but also requires substantial prior data and is less effective for novel polymorphisms. As the number of loci increases, the per-SNP costs can exceed those of the arrays.

3. **Reduced representation sequencing (RRS)** targets genome subsets for variant detection without requiring prior genomic data (Delseny *et al.* 2010, Van Tassell *et al.* 2008). Typical implementations include genotyping-by-sequencing (GBS) (Elshire *et al.* 2011), RAD-Seq (Davey and Blaxter 2010), ddRAD-Seq (Peterson *et al.* 2012), GRAS-Di (Hosoya *et al.* 2019), and MIG-Seq (Suyama and Matsuki 2015). These methods are cost-effective for large sample sets and widely used in plant breeding.

RRS provides practical advantages in citrus plants where diverse plant materials are used for breeding. However, unlike arrays or multiplex amplicon sequencing, which consistently target defined regions, RRS methods yield varying SNP sets that depend on the methodology and plant material. This variability makes it difficult to verify or compare specific loci across datasets, thereby limiting cross-platform comparisons and the reuse of results. Thus, a genotyping and data-processing approach that combines low cost and minimal prior data requirement of RRS with the locus consistency of target-defined genotyping methods is needed.

### Demand for unified genotype data

Genome-wide genotype data have been generated in citrus breeding at NARO using various technologies, including bead arrays (Illumina GoldenGate Assay) and RRS methods, such as GBS, ddRAD-Seq, and GRAS-Di, depending on the target and population. As data from indigenous cultivars, hybrids, and selected lines have accumulated, integrating these datasets has proven challenging, owing to differences in the target SNPs among platforms. These inconsistencies hinder the direct comparison of genotypes of identical SNPs. For example, a genomic prediction model built using bead array data cannot be directly applied to samples genotyped using RRS. Similarly, the lack of verifiable common SNPs limits the reuse of trait prediction models across populations and complicates model updates with new phenotype-genotype data. This also hinders GWAS follow-up, and verifying whether a genomic region strongly associated with a trait in one study exhibits the same effect in another dataset using a different marker system is often impractical. This is because the genomic positions of the SNPs obtained via RRS analysis vary depending on the method and materials used.

With declining sequencing costs, whole-genome resequencing data for many citrus cultivars, varieties, and hybrids have become available. By complementing RRS-derived genotype data from resequencing, researchers can construct a unified genotype dataset of common loci. These datasets enable the application of genomic prediction models to other sample sets and improve prediction accuracy. Additionally, by associating these defined loci with the proposed pure citrus species using ancestry informative markers (AIMs), which exhibit distinct allele patterns among ancestral species, it is possible to strengthen target gene discovery via GWAS by linking trait-associated loci to their ancestral origins.

In this study, we present an Augmented Estimation of Unified Genotype (AEUG) workflow, which converts genotypes from RRS analyses into unified genotypes at predefined loci using a reference genotype panel.

## Materials and Methods

### Plant materials

Citrus accessions, hybrid cultivars, and selected citrus lines from hybrid seedlings maintained in the NARO Citrus Breeding Program were used for both resequencing (Table 1, Supplemental Table 1) and GRAS-Di analyses (Supplemental Table 2). Those citrus accessions have been revealed to be spontaneous hybrids of ancestral mandarins and pummelos (Ollitrault *et al.* 2020, Shimizu and Nonaka 2025). The hybrids and selections/lines were the offspring of the citrus accessions (Shimizu 2022). Genomic DNA was extracted using an Illustra Nucleon Phytopure kit (Cytiva, MA, USA) according to a modified protocol (Shimizu *et al.* 2016a).

### Short-read sequencing and public data retrieval

Short-read sequencing was performed using the HiSeq 2000, 2500, and 4000 platforms, the NovaSeq/NovaSeq X systems (all from Illumina), and the DNBSEQ platform (MGI). Paired-end reads of 101 or 150 bp were generated for each sample, yielding 59–119 million read pairs per sample (13.6–35.7 Gbp; Supplemental Table 1). Short-read sequences of *Citrus*, *Fortunella* (kumquat), and *Poncirus* (trifoliata orange) species were also obtained from the public Short Read Archive (SRA) (https://www.ncbi.nlm.nih.gov/sra/). Their classifications were based on metadata annotations available in the archive. The average number of paired-end reads in the downloaded data was 69.6 million, corresponding to 18.5 Gbp.

### Mapping and variant calling of short-read sequences

Short-read analysis was conducted using a Linux PC (Debian 12; https://www.debian.org/). Adapters were removed and quality trimming was performed using Trimmomatic v0.39 (Bolger *et al.* 2014), and read pairs with ≥17% of their sequence trimmed were excluded from downstream analysis. The Clementine haploid genome sequence v1.0 (https://phytozome-next.jgi.doe.gov/info/Cclementina_v1_0) served as the reference genome, and only reads mapped to the nine major scaffolds (scaffold_1 to scaffold_9) were retained for variant calling. FASTQ reads were mapped using GSNAP (version 2021-12-17) (Wu *et al.* 2016) using the option --max mismatches = 0.125. The resulting BAM files were coordinate-sorted, and PCR duplicates were removed using Picard Toolkit v1.138 (Broad Institute, https://broadinstitute.github.io/picard/). Variant calling for each sample was performed using HaplotypeCaller in GATK 4 (https://gatk.broadinstitute.org) (McKenna *et al.* 2010) with the options -ERC GVCF and --dont-use-soft-clipped-bases T to produce GVCF files. Subsequently, the combined GVCFs of GATK4 were used to merge GVCF files from the target samples, and GenotypeGVCFs converted the merged data into VCF format.

### GRAS-Di sequencing and variant calling

Genomic DNA (500 ng) was submitted to GeneBay Co., Ltd. (Yokohama, Japan) for sequencing library construction and Genotyping by Random Amplicon Sequencing-Direct (GRAS-Di) analysis following the recommended protocol. GRAS-Di sequencing was performed using a HiSeq 4000 system (Illumina), producing 150 bp paired-end reads at an average 5.9 million reads (1.8 Gb) per sample.

BAM files were coordinate-sorted using the Picard Toolkit, and secondary alignments, supplementary alignments, and unmapped reads were removed using SAMtools (version 1.14; options -b -F 0 × 904) (Danecek *et al.* 2021). Variant calling was then performed by converting the data to the GVCF format with HaplotypeCaller, followed by merging all target-sample GVCF files using CombineGVCFs and converting them to the VCF format with GenotypeGVCFs.

### Variant filtering and annotation of resequencing data

The VCF file obtained from the resequencing analysis was filtered using BCFtools (v1.21) (Danecek *et al.* 2021), VCFtools (v0.1.16) (Danecek *et al.* 2011), and BEDTools (v2.30.0) (Quinlan and Hall 2010) to select valid genotypes. Among the called variants, we first filtered with BCFtools (-g 5) to exclude sites within 5 bp of indels, followed by filtering with VCFtools (--minDP 10) to remove genotypes with DP<10 and selected biallelic SNPs. Subsequently, we excluded sites with missing alleles with BCFtools and removed sites with an average depth ≥200 with VCFtools (--max-meanDP 200). Finally, we applied quality thresholds with the following criteria: QD<2, FS>60, SOR>3, MQ<38, MQRankSum<-7, ReadPosRankSum<-8, BaseQRankSum<-6 or BaseQRankSum>6. Mendelian violations (MVs) in known parent–child trios were detected using the Picard Toolkit, and affected sites were excluded. Variants with minor allele frequency (MAF) ≥0.01 were retained for downstream analysis. The final filtered VCF file was annotated using SnpEff software (version 5.2f) (Cingolani *et al.* 2012).

### Filtering and preprocessing of GRAS-Di genotype data

The VCF files generated from the GRAS-Di analysis were filtered using the same method as the resequencing data, following the procedure outlined below. After excluding sites within 5 bp of the indels, we used VCFtools (--minDP 8) to remove sample genotypes with DP<8 and retained only biallelic SNPs. Subsequently, variants were filtered based on the mapping quality thresholds (MQ<39 or MQ<40), and sites were excluded if they met any of the following criteria: QD<2, FS>60, SOR>3, MQRankSum<-5, ReadPosRankSum<-5, BaseQRankSum<-4, or BaseQRankSum>4. The VCF files filtered by the MQ conditions were further filtered by applying four thresholds for F_MISSING scores (0.7, 0.5, 0.2, or 0.1), resulting in sparse sample genotype dataset files (S-VCFs) defined by eight filtering conditions. Before using the S-VCF files for genomic prediction (GP) and GWAS, missing genotypes were imputed using Beagle (v5.4) (Browning *et al.* 2018).

### Selection of AIMs based on principal component analysis (PCA)

The PCA of the VCF file was performed using PLINK2 (v2.0.0a3.5; https://www.cog-genomics.org/plink/2.0/) (Chang *et al.* 2015). The resulting eigenvec file was imported into R (version 2025-04-11) (R Core Team 2023) within the RStudio integrated development environment (2025.05.0 Build 496) (Posit Team 2025) and visualized using the ggplot2 package (Wickham 2016).

The selection of AIMs (Rosenberg *et al.* 2003), which are genetic variants that exhibit large allele frequency differences among populations and thus can be used to infer ancestry (Bidyananda *et al.* 2024), was based on both the PCA results and the estimated heterozygosity from the reference genotype panel (R-VCF) genotypes. Three to four candidate plants were selected as representatives of each citrus species. Species-specific loci were identified as sites where all candidate plants belonging to a particular species were homozygous (REF or ALT). Loci shared by multiple species, regardless of the allele state (REF or ALT), were removed, retaining only those unique to a single species. The final set of loci, defined by their REF and ALT alleles, represented markers specific to each assumed citrus species.

### Selection of reference genotype panel for AEUG workflow

We evaluated the genetic similarity among all samples in the VCF file obtained from the resequencing analysis using the BCFtools gtcheck-e 0, which calculates the percentage of mismatches (MM score) across all loci. Samples with an MM score of ≤0.6% were regarded as genetically identical; one representative was retained and the others were excluded from further analysis. After removing duplicates, the sample names in the VCF file were replaced with serial IDs to prevent duplications during subsequent analyses. The resulting VCF file served as the R-VCF.

### VCF integration and accuracy assessment of genotype imputation in the AEUG workflow

Eight S-VCFs obtained from the GRAS-Di analysis under eight filtering conditions (MQ>39 or MQ>40, combined with F_MISSING thresholds of 0.7, 0.5, 0.2, or 0.1) were merged with the R-VCF, excluding genetically redundant samples, using the BCFtools merge command. The merged VCF files were imputed using Beagle (v5.4). After imputation, only the loci present in the S-VCFs were removed. Next, the genotype calls for the R-VCF samples were dropped while retaining the loci. Finally, any loci fixed as homozygous REF or ALT across all remaining samples were excluded. The remaining loci were filtered using a MAF threshold of 0.03. The annotations from the original R-VCF were copied to the filtered VCF to produce the final unified sample genotype dataset files by AEUG workflow (A-VCFs).

Imputation accuracy was assessed using 20 samples common between the S-VCF and R-VCF datasets. The percentage of genotype matches was calculated for each sample, and the R-VCF genotypes served as the reference and the A-VCF genotypes as the imputed data.

### Validation of AEUG workflow through genomic prediction and GWAS for fruit traits

Fruit trait data—fruit weight (FruW), appearance (Appear), fruit shape (Shape), fruit hardness (FruH), rind color (FruC), pericarp smoothness (Pericarp), ease of peeling (Peeling), aroma intensity (Aroma), flesh color (FleC), fruit hardness (FleH), juiciness (Juicy), firmness of locule membrane (SegM), number of seeds (Seed), bitterness (Bitter), taste (Taste), soluble solids content (Brix), and acidity (Aci)—were collected following previously described methods (Minamikawa *et al.* 2017).

All statistical analyses were conducted using R (R Core Team 2023) in the RStudio environment (Posit Team 2025). The VCF files were imported using the vcfR package (Knaus and Grünwald 2017).

Genomic prediction was performed using the single-kernel genomic best linear unbiased prediction (GBLUP) model implemented in the mmes() function of the sommer package (Covarrubias-Pazaran 2016). The additive relationship matrix (kinship matrix) was computed from the genotype matrix using the A.mat() function, assuming additive marker effects. A 10-fold cross-validation scheme was applied, training the model on 90% of the samples and testing the remaining 10%. In 10-fold cross-validation, the sample labels were first randomly shuffled using the sample() and rep() functions with a specified random seed, and then grouped into the designated number of subsets (10 folds in this case) using the split() function. Using the shuffled dataset, 10-fold cross-validation was performed 10 times with different random seeds to obtain the mean and standard error (SE) for comparison. In each fold, additive genetic effects of the training individuals were estimated, and the genomic estimated breeding values (GEBVs) of the test individuals were predicted based on the additive kernel that links the training and test sets. An MAF threshold of 0.05 was applied.

For GWAS, the RGWAS.normal function in the RAINBOWR package (Hamazaki and Iwata 2020) was used, with significant SNPs identified using the “BH” method at false discovery rate (FDR) thresholds of <0.05 or <0.01. The plots were visualized using the ggplot2 (Wickham 2016) and ggsci (Xiao 2024) packages. Quantile–quantile (QQ) plots were generated by comparing the observed and expected –log_10_ (*p*) values from the GWAS results to evaluate the distribution of test statistics and detect deviations from the null expectation.

## Results

### AEUG Workflow

**Fig. 1.**
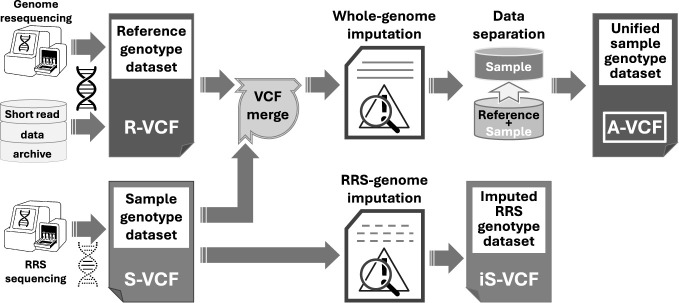
Process of the Augmented Estimation of Unified Genotype (AEUG) workflow. RRS, reduced representation sequencing (GRAS-Di in this study); VCF, variant call format; R-VCF, reference genotype panel from resequencing analysis; S-VCF, sample genotypes from GRAS-Di (not imputed); iS-VCF, imputed S-VCF; A-VCF, S-VCF converted with the AEUG workflow.

**Fig. 2.**
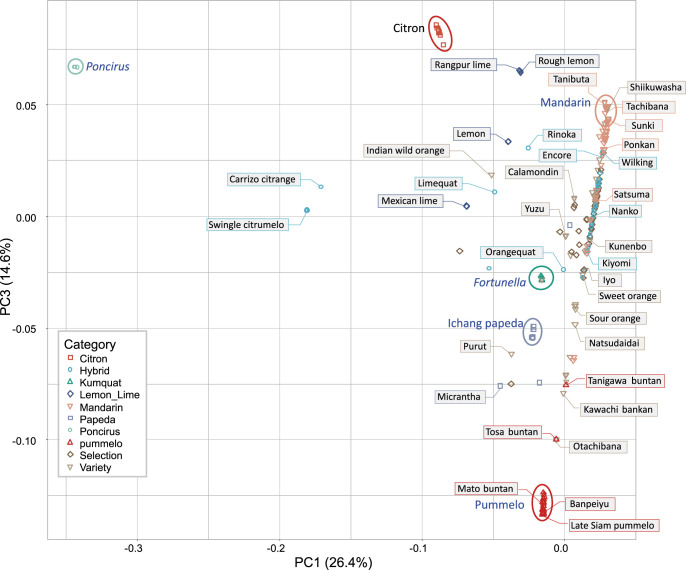
PCA plot of 322 plant samples from resequencing analysis (PC1 vs. PC3). Symbols: red square, citron; cyan circle, hybrid; blue triangle, *Fortunella* (kumquat); blue diamond, lemon and lime; orange inverted triangle, mandarins; blue square, papeda; light green circle, *Poncirus*; red triangle, pummelo; brown diamond, selections; pale brown inverted triangle, indigenous varieties/cultivars. Circles indicate clusters for pure species: mandarin (*C. reticulata*), pummelo (*C. maxima*), citron (*C. medica*), Ichang papeda (*C. cavaleriei*), *Poncirus* (*Poncirus* spp.), and *Fortunella* (*Fortunella* spp.).

**Fig. 3.**
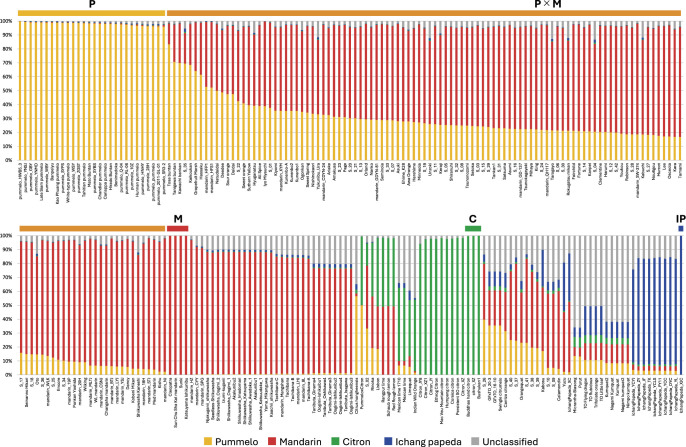
Genome composition plots of 250 R-VCF samples using estimated ancestry informative markers for four pure species. Colors: yellow, pummelo; red, mandarin; green, citron; blue, Ichang papeda; gray, unclassified. Vertical axis: composition ratio (%). Genetically identical individuals were excluded.

**Fig. 4.**
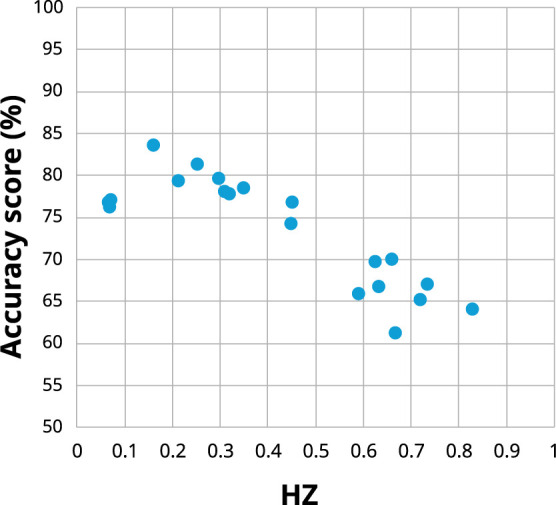
Correlation between imputation accuracy and heterozygosity in whole-genome imputation. Average heterozygosity (HZ) and imputation accuracy for 20 samples shared by R-VCF and iS-VCF. Scores represent the average across eight datasets.

We developed an AEUG workflow to integrate genotype datasets obtained from different marker platforms into a unified set of predefined loci. The workflow begins by selecting genome-wide genotypes from whole-genome resequencing data of diverse citrus accessions, hybrids, and selected lines (Table 1) to construct a reference genotype panel (Fig. 1). Genome-wide genotypes obtained from RRS methods, such as GRAS-Di, were combined with the reference panel and subjected to whole-genome imputation. A unified genotype dataset for the target samples was extracted from the imputed data and used for downstream analysis (Table 2, Figs. 2–4).

**Fig. 5.**
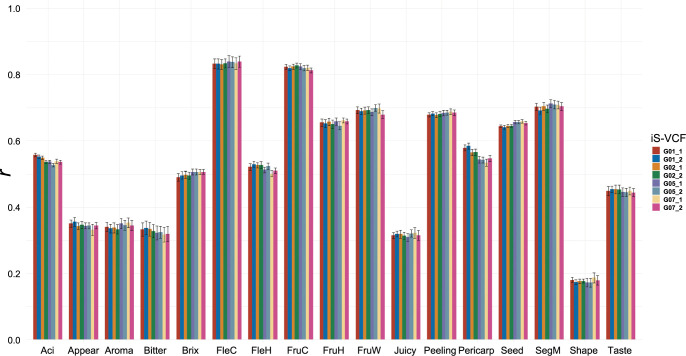
Genomic prediction of 17 fruit traits using the imputed GRAS-Di genotype dataset (iS-VCF). 10-fold cross-validation was performed ten times with different random seeds for each of the eight datasets. The bar height represents the mean prediction accuracy for each dataset, and the error bars indicate the standard error (SE) across the ten trials.

**Fig. 6.**
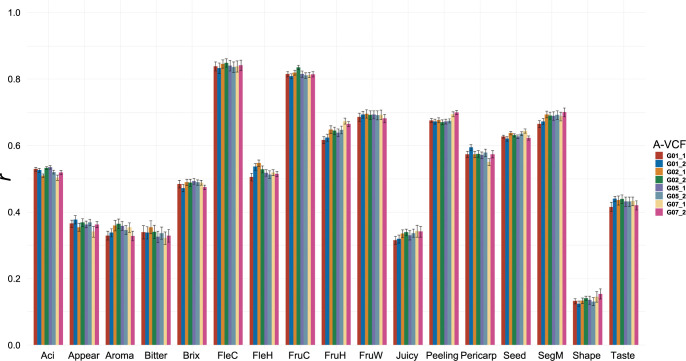
Genomic prediction of 17 fruit traits using the A-VCF dataset converted from the GRAS-Di genotype dataset by the AEUG workflow. See Fig. 5 for details.

**Fig. 7.**
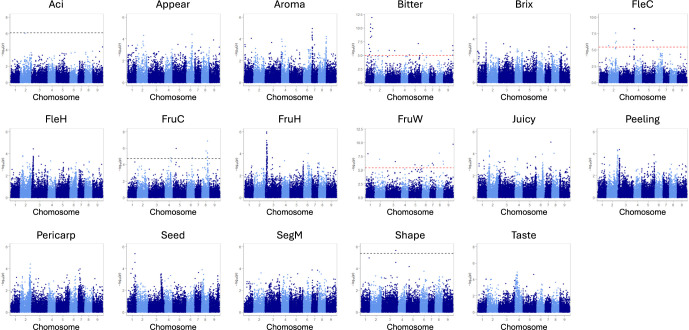
GWAS plot of 17 fruit traits using the G02_2 iS-VCF genotype dataset. Horizontal dashed lines indicate significance thresholds calculated using FDR control (black: *p* = 0.05; red: *p* = 0.01).

**Fig. 8.**
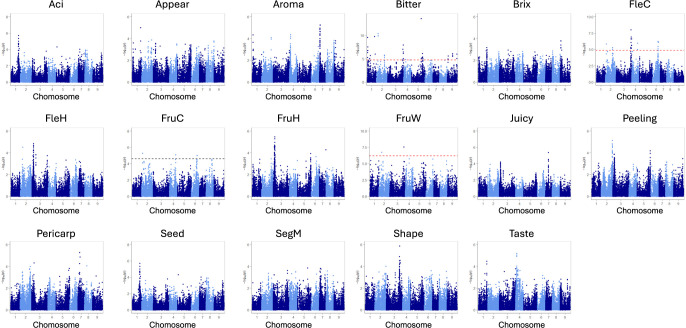
GWAS plot of 17 fruit traits using the G02_2 A-VCF genotype dataset. See Fig. 7 for details on the horizontal dashed line.

We evaluated the performance of the AEUG workflow by comparing the genomic prediction accuracy and GWAS results obtained from unified genotypes with those obtained from the original RRS genotypes. Both prediction accuracy (Figs. 5, 6) and significant SNP detection power were comparable between the two datasets (Figs. 7, 8). In whole-genome imputation, the genetic similarity between the reference panel and target samples, as well as the minor allele frequency in the reference panel, are the primary determinants of imputation accuracy (Zhang *et al.* 2022). As low-quality imputation can lead to false positives in GWAS, assessing the genetic relationships between the reference and target is essential.

To explore potential applications of AEUG workflow, we examined its functionality in linking genomic information to citrus ancestry by selecting AIMs (Table 3). The associated alleles from a unified dataset of these pure ancestral species, thereby extending the potential utility of the reference panel for dissecting genomic diversity (Tables 4, 5).

### Generation of the reference genotype dataset by resequencing analysis

The AEUG workflow comprises four main steps (Fig. 1):

1. Collecting reference genotype datasets from genetically related materials.

2. Obtaining genotype data from RRS of target samples.

3. Merging reference and sample datasets, followed by whole-genome imputation.

4. Extracting target sample genotypes aligned to the reference positions (unified sample genotype dataset).

First, an R-VCF was constructed from resequencing data of plants genetically close to the target samples. This panel was merged with the S-VCF obtained using RRS technology (GRAS-Di in this study). Missing genotypes were imputed, and the genotypes of the target samples at the R-VCF loci were extracted as A-VCF.

The reference panel included short-read data from 126 citrus genetic resources, hybrids, and selected lines maintained by NARO, including known pummelos, mandarins, and kumquats (Table 1). Additionally, short-read data for 196 citrus plants and related species from the SRA, including native varieties such as pummelo, mandarin, citron, lemon, lime, and Ichang papeda; hybrids, as well as trifoliate oranges and kumquats, were incorporated, producing 322 accessions for variant calling. To minimize imputation errors, loci with missing genotypes were excluded prior to whole-genome imputation. To exclude imputation errors, loci containing missing genotypes were first removed. Additionally, 0.041% of loci showing Mendelian violations in 35 reference trios were excluded. This resulted in a VCF comprising 779,247 SNPs across 322 samples (Table 2A). The average variant density across the 9 major scaffolds was 370 variants per 100 kb, peaking on scaffold_5 (449) and the lowest on scaffold_4 (311) (Table 2A).

Based on functional annotation with snpEff, 12.38% of the polymorphisms were located in exons, 22.73% in introns, and 10.92% in intergenic regions (Table 2B). Approximately one-third of the variant loci were located within the gene regions. Loci upstream and downstream of genes accounted for 21.17% and 28.44%, respectively, while ˂5% were in 5ʹ or 3ʹ untranslated regions (UTRs).

### Validation of the population structure of reference samples using PCA

The PCA plot of the merged VCF genotypes showed that indigenous cultivars, such as Satsuma, sweet orange, sour orange, Ponkan, natsudaidai, and Iyo, together with crossbred hybrids, including Kiyomi, Nanko, Encore, and Wilking, were positioned along the axis connecting clusters of indigenous mandarins (Tachibana, Tanibuta, Shiikuwasha, and Sunki) (Wu *et al.* 2021) and pummelos (Banpeiyu, Mato Buntan, and Late Siam pummelo) (Fig. 2). Major cultivars derived from interspecific hybridization involving sweet orange, Satsuma, and Ponkan with other indigenous cultivars or hybrids did not form distinct clusters, but were continuously distributed (Fig. 2, Supplemental Fig. 1).

The citron (*C. medica*) and Poncirus clusters were located distant from the mandarin and pummelo clusters. Lemon, a hybrid of sour orange and citron (Curk *et al.* 2016, Talon *et al.* 2020), was positioned approximately midway between the citron cluster and sour orange. Carrizo citrange (*Poncirus* × sweet orange) and Swingle citrumelo (*Poncirus* × grapefruit) were located midway between the *Poncirus* cluster and sweet orange or grapefruit, respectively, which was consistent with their documented hybrid origins (Swingle and Reece 1967).

The *Fortunella* (kumquat) cluster appeared in an intermediate position, slightly deviating from the line connecting the mandarin and pummelo clusters. Calamondin (*Fortunella* × unknown mandarin), a natural kumquat hybrid, and Orangequat (Satsuma × [*Fortunella japonica* × *F. margarita* “Meiwa”]) (Swingle and Reece 1967) were positioned as expected from their parentage (Fig. 2). Ichang papeda (*C. cavaleriei*, syn. *C. ichangensis*) and Micrantha (*C. micrantha*, syn. *C. hystrix*), both regarded as pure citrus species (Ollitrault *et al.* 2020), occupied positions distinct from the mandarin, pummelo, citron, *Poncirus*, and *Fortunella* clusters. “Purut”, a native citrus genetic resource of uncertain origin thought to be *C. hystrix*, was placed separately from *C. micrantha*.

Overall, these results demonstrate that the resequencing dataset captures sufficient genetic diversity and polymorphic allele information to enable accurate imputation for hybrids between mandarin and pummelo, which are common in citrus breeding (Shimizu and Nonaka 2025). Furthermore, although their use in breeding is limited, the dataset also appears valuable for evaluating hybrids involving citron, Ichang papeda, kumquat, and trifoliate orange.

### Validation of diversity and genomic structure in the citrus reference genotype panel

To achieve high-precision genomic breeding by associating the origins of important genes with ancestral species, we identified AIMs. These markers have been widely used in studies on population structure, phylogeography, and admixture mapping across various organisms, including plants, to trace lineage relationships and identify genomic regions associated with specific ancestral backgrounds (Bidyananda *et al.* 2024).

Citrus materials were selected from the merged VCF that were closely related to pure citrus species, with the aim of elucidating the genomic relationships among the proposed pure citrus species, natural hybrid cultivars, and crossbred cultivars. Based on the PCA results of the merged VCF (322 samples) and available database annotations, we selected candidate plants representing six pure citrus species: citron, pummelo, mandarin, Ichang papeda, *Fortunella*, and *Poncirus*. Micrantha and Purut, both classified as *C. hystrix*, were excluded owing to limited data and their distant positions in the PCA plot. A sufficient number of plants annotated as Ichang papeda were retained in the Ichang papeda group. For each species, candidates were selected based on the lowest heterozygosity scores in the merged VCF and were assumed to represent pure genotypes. Unique AIM loci and alleles were identified in each of the selected species.

The merged VCF contained genetically equivalent samples, such as sports, nucellar seedlings, and synonyms, which could distort allele frequency estimates and introduce errors during whole-genome imputation (Zhang *et al.* 2022). To mitigate this, we removed 95 samples from the 39 groups, retaining 250 distinct samples. We identified 722,223 polymorphic loci (2.95–13.94% loci phased) that were used as an R-VCF for downstream analyses.

We attempted an admixture analysis of R-VCF using randomly sampled genotypes, but no evident peak was detected for *K* values ranging from 2 to 35 (data not shown). Instead, we examined allele frequency at loci specifically associated with four assumed pure species, citron, pummelo, mandarin, and Ichang papeda, excluding *Fortunella* and *Poncirus*, which belong to different genera. This resulted in 145,834 loci (20.2% of the R-VCF) assignable to one of the four species (Table 3), representing a robust set of AIMs for differentiating these ancestral backgrounds. The allelic composition analysis of the 250 R-VCF samples showed that 27 were almost entirely pummelo-derived, whereas 4 were predominantly mandarin-derived (Fig. 3). Consistent with the PCA results, cultivars such as Satsuma, sweet orange, sour orange, and grapefruit, and hybrids such as Kiyomi were derived from interspecific hybridization between mandarin and pummelo. The proportion of mandarin- versus pummelo-derived alleles showed continuous variation, indicating that these cultivars originated from recurrent interspecific hybridization of ancestral mandarin and pummelo (Talon *et al.* 2020). Lemon and lime carried both the mandarin and citron genomes, which is consistent with their proposed origin in sour orange (Curk *et al.* 2016, Talon *et al.* 2020).

Among the Ichang papeda samples, nine (excluding one, Ichang papeda XC, likely a mandarin hybrid) contained approximately 17% of the genome of unknown origin. As our ancestry estimation did not include diversity within *Fortunella* or *Poncirus*, their genomes likely contributed to the unclassified fraction. A small proportion of alleles from the four pure species was also detected in *Fortunella* and *Poncirus*, but these may be artifacts of the estimation method. Overall, these results suggest that species assignments outside the genus *Citrus* may be less reliable.

### Evaluation of AEUG workflow performance under different variant filtering conditions

In this study, we selected 192 materials for GRAS-Di analysis, including 12 indigenous cultivars, 5 known mandarins, 4 pummelo, 48 crossbred cultivars, and 123 selected lines (Table 4). As indicated by the PCA (Fig. 2), the crossbred cultivars and selected lines were hybrids of mandarin and pummelo. These results are consistent with the current understanding of the domestication and diversification process of citrus varieties, in which indigenous cultivars arose through repeated hybridizations between ancestral mandarins and ancestral pummelos. They share common ancestries, indicating that these materials—including crossbred hybrids and selections/lines—retain common haplotypes.

To evaluate the effect of variant calling thresholds on genomic prediction and GWAS results, we applied four F_MISSING thresholds (0.7, 0.5, 0.2, and 0.1) and two minimum mapping quality (MQ) thresholds (39 and 40) to produce eight VCF datasets (S-VCFs). Genome coverages of mapped GRAS-Di reads were 2.48–5.08% (mean 3.65%), and read depths were 43.9–81.4 (mean 56.4) for 192 samples. An increased F_MISSING threshold facilitates more missing data per locus, whereas high MQ thresholds increase the reliability of the called variants (40 is generally recommended). As expected, the SNP counts increased with increasing F_MISSING and decreased with increasing MQ, ranging from 23,097 (G01_2) to 68,458 (G07_1) loci (Table 5).

Each S-VCF was converted to a unified genotype dataset using the AEUG workflow. Specifically, it was merged with the reference genotype panel (R-VCF; 250 samples, 722,223 loci, no missing data), imputed with Beagle v5.4, stripped of loci unique to S-VCF, and the R-VCF reference sample genotypes were removed (4.17–6.28% of S-VCF loci shared with R-VCF). Loci in which all samples were homozygous for REF or ALT were excluded, followed by MAF filtering to yield the final A-VCFs.

The number of loci converted from the S-VCF increased proportionally with the original S-VCF size, from 122,340 (G01_2) to 190,028 (G07_1) before MAF filtering, and 65,707 to 85,603 loci after applying MAF ≥0.03 (Table 5). These accounted for 9.10–11.85% of the loci in R-VCF. This indicates that large S-VCFs improve genotype inference by whole-genome imputation even with high missing rates.

The imputation accuracy was evaluated for 20 samples shared between the S-VCF and R-VCF datasets. The accuracy was calculated as the percentage of matching genotypes between A-VCF and R-VCF, with R-VCF as a reference. Across the eight datasets, the mean accuracy ranged from 71.9% to 75.0% (Supplemental Table 3), and improved slightly with a low F_MISSING threshold. The accuracy varied by sample, from 61.3% (Hyuganatsu) to 83.6% (Wilking), and showed a negative correlation with heterozygosity (HZ) (Fig. 4). Although the pummelo cultivars (Banpeiyu, Hirado Buntan, and Mato Buntan) had the lowest HZ values, their accuracy was lower than that of the Wilking cultivar (Fig. 4). The MQ threshold (39 vs. 40) had negligible effects on accuracy (Supplemental Table 3).

The SnpEff annotation showed that relaxing the filtering thresholds increased the proportion of annotated loci (Supplemental Table 4). Under all conditions, the A-VCFs contained more annotated effects across all impact and functional categories than their corresponding iS-VCFs. In contrast, the number of intergenic loci in iS-VCFs increased more than threefold from G01_2 to G07_2, whereas in A-VCFs, the increase was minimal.

### Effects of filtering thresholds and unified genotype conversion on fruit trait prediction

We compared the effects of genotype filtering thresholds and the AEUG workflow on GP accuracy using datasets from RRS and their corresponding unified genotype datasets. For GP model construction, we used indigenous citrus cultivars, crossbred hybrids, and selections for which sufficient replicates of trait data were available (Supplemental Table 2; Minamikawa *et al.* 2017). Those indigenous citrus cultivars originated from historical crosses between ancestral mandarins and ancestral pummelos, and therefore share haplotypes derived from common ancestries (Ollitrault *et al.* 2020, Shimizu 2022). As these indigenous citrus cultivars have been repeatedly used as breeding parents, they also share haplotypes with both the crossbred hybrids and the selected lines used in this study.

First, we evaluated the influence of variant selection criteria for GRAS-Di-derived genotype data on the GP accuracy for 17 fruit traits. Missing genotypes in the eight datasets, filtered under different criteria, were imputed using Beagle to generate imputed VCFs (iS-VCFs; 23,286–69,898 SNPs; all loci phased). GP accuracy was assessed by 10-fold cross-validation and compared with the results from 1,841 SNPs obtained via the GoldenGate Assay (Minamikawa *et al.* 2017). These trends were generally consistent, although some differences were observed.

Ten traits (Appear, Aroma, Brix, FruW, Juicy, Peeling, Pericarp, Seed, SegM, Taste) showed improved accuracy, with notable gains in Aroma (0.05→0.45), Juicy (0.1→0.3), and Taste (0.2→0.45) (Fig. 5) to those reported by Minamikawa *et al.* (2017). These results are consistent with previous findings that such traits are polygenic and that increasing the number of SNPs improves model accuracy. Six traits (Aci, Bitter, FleH, FruC, FruH and Shape) exhibited accuracy levels similar to those reported by Minamikawa *et al.* (2017), with only a slight decrease in FleC, likely owing to model overfitting from excessive genotype data. Shape, a visually scored trait that lacks ordinal ranking, is inherently less reliable. Across the eight filtering conditions, the accuracy differences were minimal; however, slight decreases were observed for Aci, Appear, Aroma, Bitter, and Pericarp as the missing data increased, whereas Brix and FleC showed small gains. None of the differences were statistically significant.

Next, we compared the GP accuracies of the iS-VCFs (Fig. 5) and the unified genotype dataset (A-VCFs) generated using the AEUG workflow (Fig. 6). Ten traits (Appear, Aroma, Bitter, FleC, FleH, FruC, FruW, Juicy, Peeling and Pericarp) showed nearly identical accuracies, whereas six (Aci, Brix, FruH, Seed, SegM and Taste) showed slightly lower accuracies with A-VCFs. Shape accuracy decreased by approximately half when using A-VCFs, again indicating its scoring limitations. The standard errors after 10 trials were sufficiently small for both iS-VCF and A-VCF across all traits and datasets, indicating that any bias introduced by trait data partitioning and resampling during 10-fold cross-validation was negligible (Figs. 5, 6).

The prediction accuracy varied slightly among A-VCFs than among iS-VCFs, likely because the AEUG workflow performs imputation and locus conversion simultaneously, which may increase imputation errors. Minor decreases in some traits may also indicate overfitting from larger SNP sets and redundant markers, which act as noise. However, the differences in accuracy between the iS-VCFs and A-VCFs were negligible for all traits, indicating that the AEUG workflow is suitable for genomic prediction. The scatter plots and regression lines of genomic predicted values versus observed values obtained from 10-fold cross-validation using iS-VCF and A-VCF were nearly identical across all 17 fruit traits (Supplemental Figs. 2, 3), further confirming the effectiveness of the AEUG workflow for genomic prediction applications.

### Comparable GWAS performance of A-VCF and its utility in breeding via ancestral genome mapping

GWAS were conducted on 17 fruit traits using the G02_2 dataset. The iS-VCF and A-VCF datasets contained 32,284 and 72,869 SNPs, respectively.

In the GWAS based on the iS-VCF dataset, significant association signals above the genome-wide threshold were detected for five traits: fruit weight (FruW), fruit shape (Shape), rind color (FruC), flesh color (FleC), and bitterness (Bitter) (Fig. 7). However, for FruW and Shape, the peaks were frequently associated with a single SNP, indicating possible false associations. Although not exceeding the threshold, peaks associated with multiple SNPs were observed for fruit hardness (FruH) and flesh hardness (FleH).

In the GWAS based on the A-VCF dataset, significant SNPs above the threshold were detected for four traits: FruW, FruC, FleC and Bitter (Fig. 8). As in the iS-VCF results, suggestive SNPs for FruH and FleH appeared in the same genomic regions, but were below the significance threshold. For Aroma, both datasets showed regions supported by multiple SNPs at similar positions, although the differences were not statistically significant. Common multi-SNP peaks between iS-VCF and A-VCF were observed for FruC and FleC. The Quantile–quantile (QQ) plots for 17 traits using iS-VCF (Supplemental Fig. 4) and A-VCF (Supplemental Fig. 5) indicated that the selected GWAS model was statistically robust and reliable, showing no systematic bias or inflation in test statistics. The results were consistent with those obtained from the Manhattan plots, confirming that the FDR thresholds were appropriately set.

For acidity (Aci), iS-VCF showed a single SNP peak marginally below the threshold, likely indicating a false association. However, in A-VCF, a subthreshold peak on chromosome 1 was supported by multiple SNPs and was regarded as a potential candidate region. Overall, the reproducibility between datasets was low for single SNP peaks, which were likely false positives resulting from locus conversion and imputation in the AEUG workflow. In contrast, peaks supported by multiple SNPs and reproduced in both datasets, such as FruH, FleH, Aroma and FleC, were considered candidate genomic regions associated with the target trait. These findings indicate that even with moderate imputation accuracy in AEUG workflow, GWAS can detect trait-associated regions when supported by multiple SNPs.

To further assess the utility of A-VCF in genomic studies, we examined whether it could link genotypic data to ancestral citrus genomes. Using the G07_2 dataset, loci were compared with those specific to four assumed pure species: mandarin, pummelo, citron and Ichang papeda. In the iS-VCF dataset, only 620 SNPs (1.01% of 61,139) matched the loci of these species, limiting the ancestral genome analysis. In contrast, the A-VCF dataset contained 31,438 matching SNPs (36.6% of the 85,877 total).

As shown in Fig. 3, many cultivars, hybrids and selected lines in the NARO citrus breeding program retained genomic segments from mandarin and pummelo. Phylogenetic analysis suggested that the introgressed regions of both species contributed to the modern fruit traits. Mapping significant SNPs to the genomes of these four pure species may facilitate the gene discovery of target traits and support marker-assisted selection strategies.

## Discussion

Genotyping of defined SNP loci using array-based or amplicon sequencing technologies provides high reliability but requires extensive prior genome sequencing and genetic diversity with allele frequency for the target crop or population, and redesigning arrays is not straightforward. These constraints limit their application in citrus seedling selection, where broad genomic diversity must be considered.

In contrast, advances in sequencing have enabled RRS methods such as GBS (Elshire *et al.* 2011), RAD-Seq (Davey and Blaxter 2010), ddRAD-Seq (Peterson *et al.* 2012), GRAS-Di (Hosoya *et al.* 2019), and MIG-Seq (Suyama and Matsuki 2015) to detect polymorphisms by sequencing subsets of the genome. These approaches have been increasingly applied in citrus breeding. However, short-read data from the RRS frequently shows substantial variations in read depth by sample or locus and typically contain a high proportion of missing data (Chattopadhyay *et al.* 2014, Wickland *et al.* 2017). Consequently, polymorphic loci identified by RRS vary considerably depending on the technology and target samples, complicating direct comparisons of genomic prediction models and evaluation of the contributions of specific genomic regions to traits. Heterogeneity remains a primary challenge in citrus breeding.

To address this aspect, we developed the AEUG workflow, which converts variable-locus genotype datasets from the RRS into defined-locus genotype datasets using a reference genotype panel, and validated its effectiveness. The reference panel comprised 322 citrus accessions, including major cultivars, crossbred cultivars with selected strains mostly from the NARO citrus breeding program, wild citrus species, and related genera (*Fortunella* and *Poncirus*). Using whole-genome resequencing data, a robust SNP set was selected to construct the panel. The application of AEUG workflow converted RRS-derived genotypes to reference loci genotypes and standardized SNP sets across samples and technologies, and enabled their integration into a common reference-based dataset. With moderate imputation accuracy (71.9–75.0%), the genomic prediction accuracy for 17 fruit traits remained largely unchanged before and after AEUG processing. Considering the common ancestries of the plant materials used in this study, our results are consistent with previous findings indicating that genomic prediction accuracy is driven primarily by the genetic relatedness between the training and target populations, rather than by minor differences in genome imputation accuracy (Alemu *et al.* 2024, Asoro *et al.* 2011). A similar approach to integrating genotype datasets from different typing systems has been reported by Minamikawa *et al.* (2024) for apple. They combined Infinium and GRAS-Di genotypes without removing the GRAS-Di loci after imputation. By increasing the number of loci through merging, they demonstrated improved genomic prediction accuracy and enhanced GWAS detection power.

In the GWAS, the profiles resembled the results by Minamikawa *et al.* (2017). However, peaks supported by a single SNP were rarely reproduced between iS-VCF and A-VCF and were likely false positives, whereas peaks supported by multiple SNPs were consistently retained, indicating robust trait associations. Additionally, whereas only a small proportion of iS-VCF loci could be linked to the four pure Citrus species, 19.5% of A-VCF SNPs were successfully assigned, providing a robust set of AIMs and enhancing the interpretation of gene–trait relationships based on ancestral genomic origin.

The SNP set obtained from resequencing effectively clarified phylogenetic relationships among *Citrus*, *Fortunella* and *Poncirus*, and a subset was associated with mandarin, pummelo, citron and Ichang papeda. These relationships are consistent with those reported in previous studies which demonstrated that most indigenous cultivars originate from recurrent hybridization between pure mandarin and pummelo species (Ollitrault *et al.* 2020, Talon *et al.* 2020), indicating no fundamental differences in the developmental processes of indigenous and crossbred cultivars. Analysis using these species-associated SNPs indicated that the major indigenous cultivars widely used in breeding resulted from repeated interspecific hybridization, particularly between mandarin and pummelo. Only 1.01% of the RRS-derived SNPs (iS-VCF) could be attributed to these species, in contrast to 36.6% in A-VCF. Notably, multiple trait-associated SNP regions detected in GWAS could be correlated with these species through the identified AIM, facilitating candidate gene identification and revealing the origins of the target traits. This is another advantage of applying the AEUG workflow, which accelerates the elucidation of the origins of significant SNPs. These results demonstrated that locus conversion via AEUG workflow, combined with knowledge of the origin of the target region, can substantially facilitate GWAS-based gene discovery.

Locus conversion via AEUG workflow transforms heterogeneous genotype datasets into unified sets of common loci, enabling a direct comparison of genomic prediction accuracies across datasets and the reuse of existing prediction models in seedling selection. This reusability is particularly valuable in fruit tree breeding, where the maintenance of populations for genetic analysis and phenotyping is time- and resource-intensive. Traditionally, the validation of trait-associated regions across populations requires the design of new DNA markers and additional genotyping. By aligning and resolving locus inconsistencies among datasets from different RRS platforms, AEUG workflow enables a more efficient and accurate evaluation of genetic gain from GWAS-detected genomic regions.

Although whole-genome imputation with Beagle 5.4 achieved accuracies of up to 83.6%, no major impact on GP or GWAS was observed even when the genotype data contained substantial missing values. Tsai *et al.* (2017) demonstrated that the inclusion of genetically related materials can substantially improve genome imputation accuracy. In our case, the plant materials used to generate the reference genotype panel share common haplotypes with those used for RSS analysis (Ollitrault *et al.* 2020, Shimizu 2022), as shown in Figs. 2 and 3, thereby contributing to higher imputation accuracy in the AEUG workflow. The use of closely related materials as training populations is also known to improve genomic prediction accuracy (Alemu *et al.* 2024, Asoro *et al.* 2011). Consistent with these reports, our results showed that even when genomic imputation accuracy in the AEUG workflow is not necessarily high, sufficient prediction accuracy for selection can be achieved when closely related materials sharing haplotypes derived from common ancestors are used to train the genomic prediction model. Therefore, when applying the AEUG workflow to other crops, it is advisable to use a sufficient number of closely related materials towards the target population to achieve higher genome-wide imputation accuracy. Incorporating closely related materials for the training population also improves genomic prediction accuracy and is beneficial in selection (Alemu *et al.* 2024, Asoro *et al.* 2011).

The proportion of missing data in the S-VCF is another point to consider that could influence GP and GWAS. In this study, whole genome imputation of a single RRS dataset (S-VCF) with Beagle 5.4 imputed all missing genotypes and estimated linkage phases in both iS-VCF and A-VCF at all loci. When alternative imputation tools are used, it is important not only to assess imputation accuracy but also to examine the proportion of estimated linkage phases and their impact on GP and GWAS.

In addition, modern citrus cultivars trace their origins to four main ancestral taxa: citron, mandarin, pummelo and papeda (Ollitrault *et al.* 2020, Talon *et al.* 2020, Wu *et al.* 2014), which are pure species. Repeated interspecific hybridization among these ancestors subsequently broadened the diversity of the genus *Citrus*. The current diversity of cultivars results from recurrent hybridization among a limited set of key ancestral plants (Shimizu *et al.* 2016b). Based on this background, the selection of AIMs was carried out using PCA and heterozygosity in this study. Future refinements may benefit from different approaches for the selection of AIMs, such as those based on *F*st with LD pruning or machine-learning strategies, to achieve higher precision.

Based on this research, we are currently integrating GP prediction models across multiple RRS datasets by converting them to a unified genotype set with the AEUG workflow. We are also comparing significant genomic regions identified in GWAS, taking AIMs into account across sample sets. Taken together, these capabilities support the broader adoption of genomics-assisted breeding in citrus and advance the goals of Citrus Breeding 2.0. These developments will be described in future publications.

## Author Contribution Statement

TS proposed the concept of the AEUG workflow and conducted DNA extraction, sequence data analysis, genetic diversity analysis with ancestor estimation, and statistical evaluation for genomic prediction and GWAS. KN managed the citrus genetic resources and hybrid populations and evaluated their fruit traits. Both TS and KN conducted genomic breeding studies based on this study. TS prepared the manuscript draft, and KN contributed to its revision. All authors have approved the manuscript for publication.

## Supplementary Material

Supplemental Figures

Supplemental Tables

## Figures and Tables

**Table 1.** Citrus samples and related taxa used for resequencing analysis

Category	Sources*^a^*	
	NIFTS	SRA
Wild species/cultivars	19	66
Pummelo	10	23
Mandarin	12	60
Citron	0	12
Lemon/lime	0	7
Papeda*^b^*	0	15
Hybrid cultivars	40	6
Selected lines	42	0
*Poncirus* spp.	0	5
*Fortunella* spp. (kumquat)	3	2
(Total)	126	196 (322)

*^a^* Source of plant or sequence data. NIFTS: Samples maintained in NIFTS. SRA: NGS data downloaded from SRA (https://www.ncbi.nlm.nih.gov/sra) were used for subsequent analyses. The sample categories of the SRA section follow the description of its data sources. *^b^* Papeda includes both *C. cavaleriei* (syn. *C. ichangensis*) and *C. micrantha*.

**Table 2.** Called variants and functional annotation of reference samples by resequencing analysis

A. Called variants with their rates for nine scaffolds		
Chromosome	Variants	Variants rate
scaffold_1	85,738	337
scaffold_2	102,873	353
scaffold_3	138,679	368
scaffold_4	82,279	311
scaffold_5	96,318	449
scaffold_6	68,120	376
scaffold_7	63,278	333
scaffold_8	64,653	388
scaffold_9	77,309	406
Total	779,247	370

B. Number of functionally annotated loci		
Type (alphabetical order)	Count	Ratio
DOWNSTREAM	640,520	28.44%
EXON	278,756	12.38%
INTERGENIC	245,834	10.92%
INTRON	489,409	21.73%
SPLICE_SITE_ACCEPTOR	418	0.019%
SPLICE_SITE_DONOR	400	0.018%
SPLICE_SITE_REGION	23,782	1.056%
UPSTREAM	476,902	21.17%
UTR_3_PRIME	74,411	3.304%
UTR_5_PRIME	21,835	0.969%
		100.0%

**Table 3.** Number of loci inferred for four assumed pure citrus species. Values in parentheses indicate the proportion of each locus to the total number of loci (722,223) detected in the 250 samples in the R-VCF dataset

Species	loci	(%)
Mandarin	35,298	4.89%
Pummelo	19,855	2.75%
Citron	63,721	8.82%
Ichang papeda	26,960	3.73%
	145,834	20.2%

**Table 4.** Composition of plant materials used for GRAS-Di genotyping analysis

Category	Samples
Varieties	12
Mandarins	5
Pummelos	4
Hybrids	48
Selections	123
	(33 families)
Total	192

**Table 5.** Polymorphic loci detected by GRAS-Di and their conversion via the AEUG workflow

Data set	F_MISSING	minMQ	Loci				
			S-VCF	S-R shared*^a^*	AEUG converted (A-VCF)		
					Imputed*^b^*	MAF = 0.03*^c^*	(%)*^d^*
G07_1	0.7	39	68,458	2,855 (4.17%)	190,028	85,603	11.85%
G07_2	0.7	40	61,139	2,824 (4.62%)	185,137	85,877	11.89%
G05_1	0.5	39	60,237	2,697 (4.48%)	180,824	82,989	11.49%
G05_2	0.5	40	54,080	2,668 (4.93%)	178,481	83,095	11.51%
G02_1	0.2	39	35,517	1,825 (5.58%)	146,196	72,597	10.05%
G02_2	0.2	40	32,284	1,801 (5.58%)	143,725	72,869	10.09%
G01_1	0.1	39	25,163	1,471 (5.85%)	127,947	66,042	9.14%
G01_2	0.1	40	23,097	1,450 (6.28%)	122,340	65,707	9.10%

*^a^* Loci and percentage of S-VCF shared with R-VCF. *^b^* Loci converted and imputed from S-VCF via the AEUG workflow prior to filtering using the MAF threshold. *^c^* Loci remaining after filtering with a MAF threshold of 0.03. *^d^* Percentage of AEUG-converted loci retained after MAF filtering relative to the total number of loci in R-VCF (722,223 loci).
