# Supplementary material for: Facilitating genomic breeding in citrus via reference panel-based whole-genome imputation of low-coverage sequencing-derived genotype data
Source: Breed Sci. 2026 Feb 18;76(1):95–110. doi: 10.1270/jsbbs.25061 (PMC13437165; doi:10.1270/jsbbs.25061)
Supplement: Supplementary file 1 [file 76_095_s1.pdf]

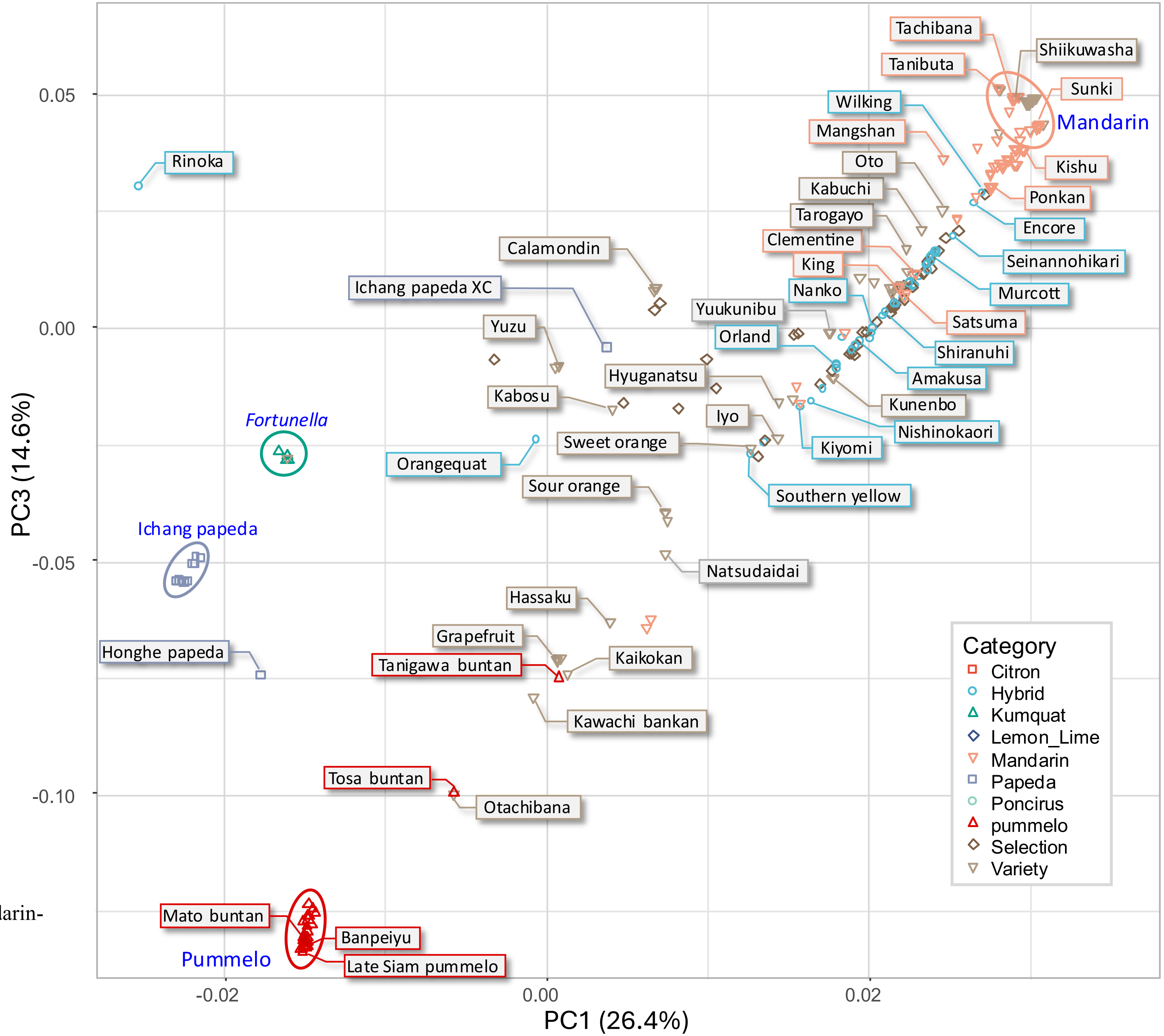

Supplemental Fig. 1.
Enlarged view around the mandarin-pummelo clusters in **Fig.2**.

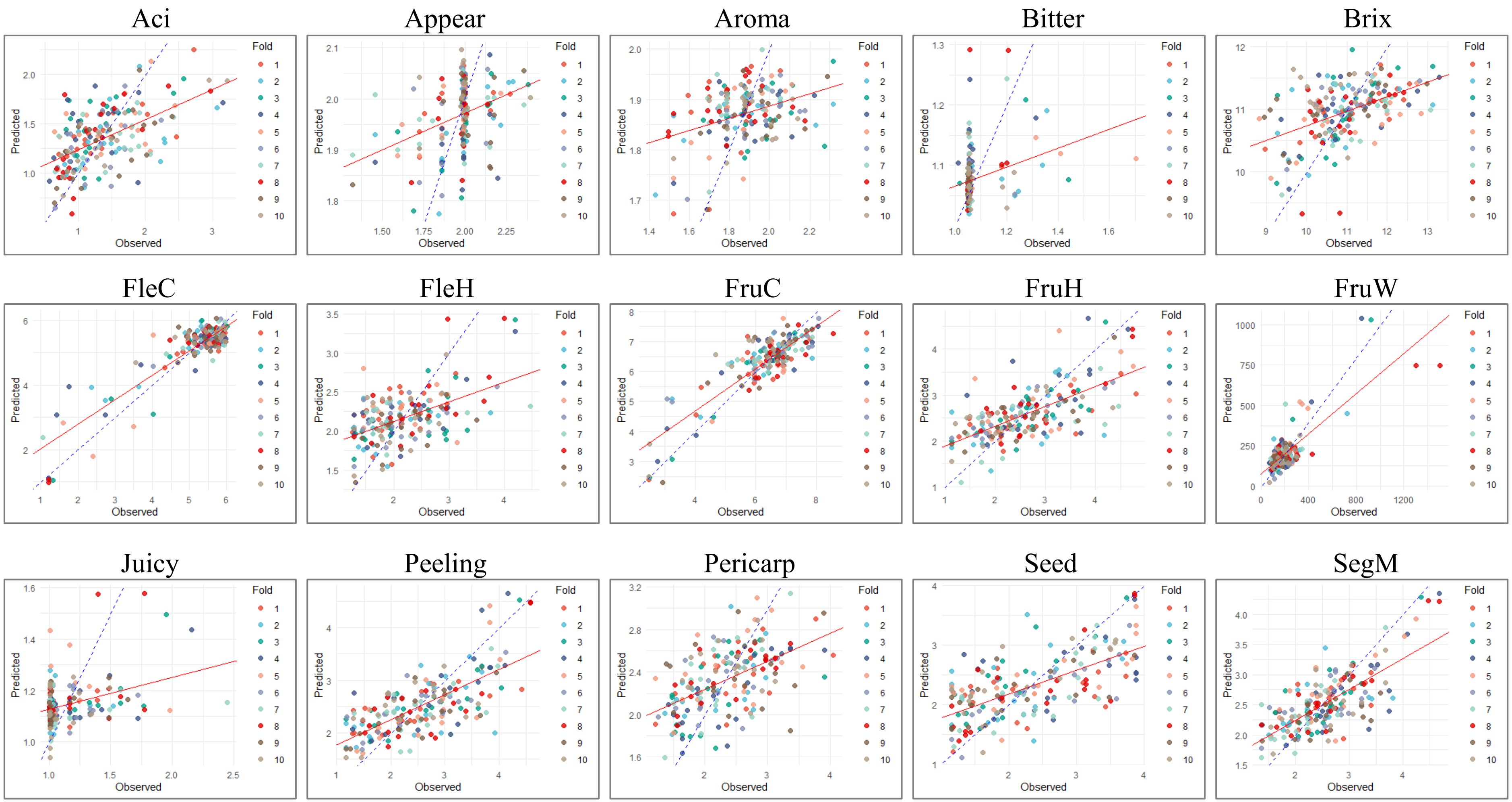

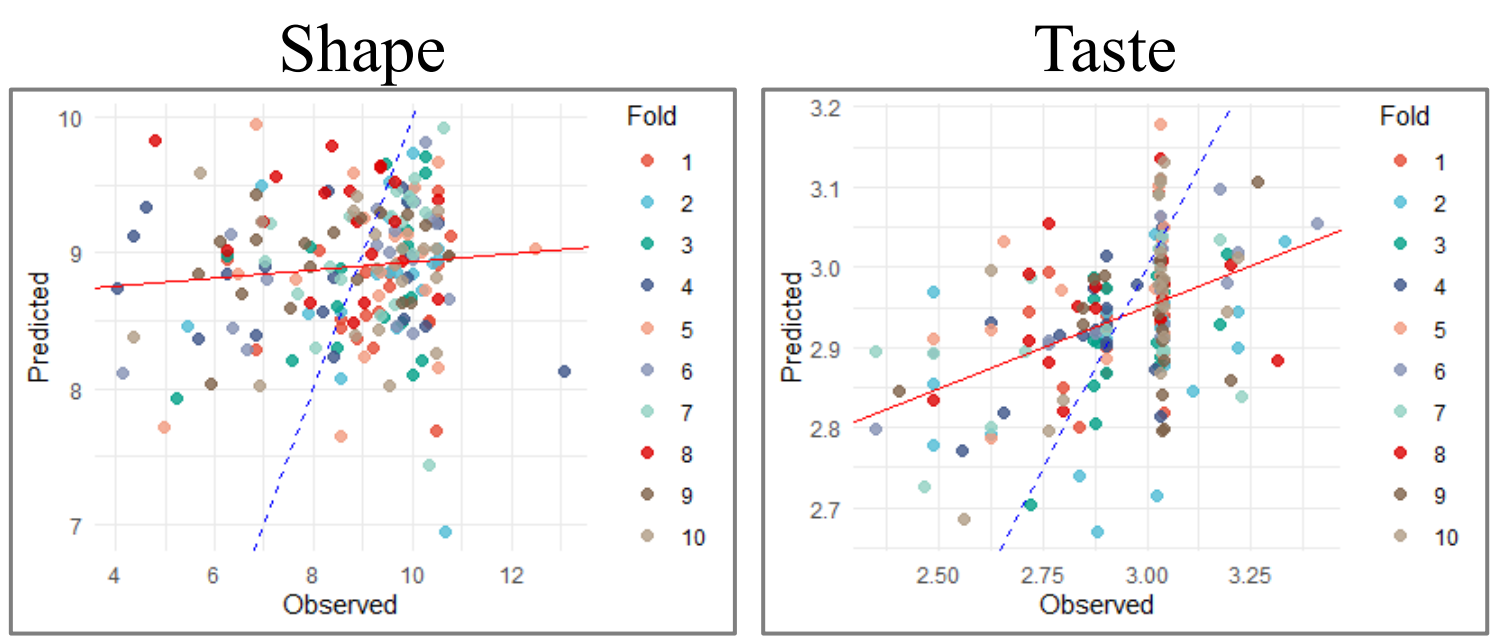

Supplemental Fig. 2. Scatter plots of observed and predicted phenotypic values obtained from 10-fold cross-validation for 17 traits using the iS-VCF genotype dataset. Each point represents an individual sample, and the dot colors correspond to the validation folds. The red solid line indicates the regression line fitted with an intercept, and the blue dashed line represents the line of identity.

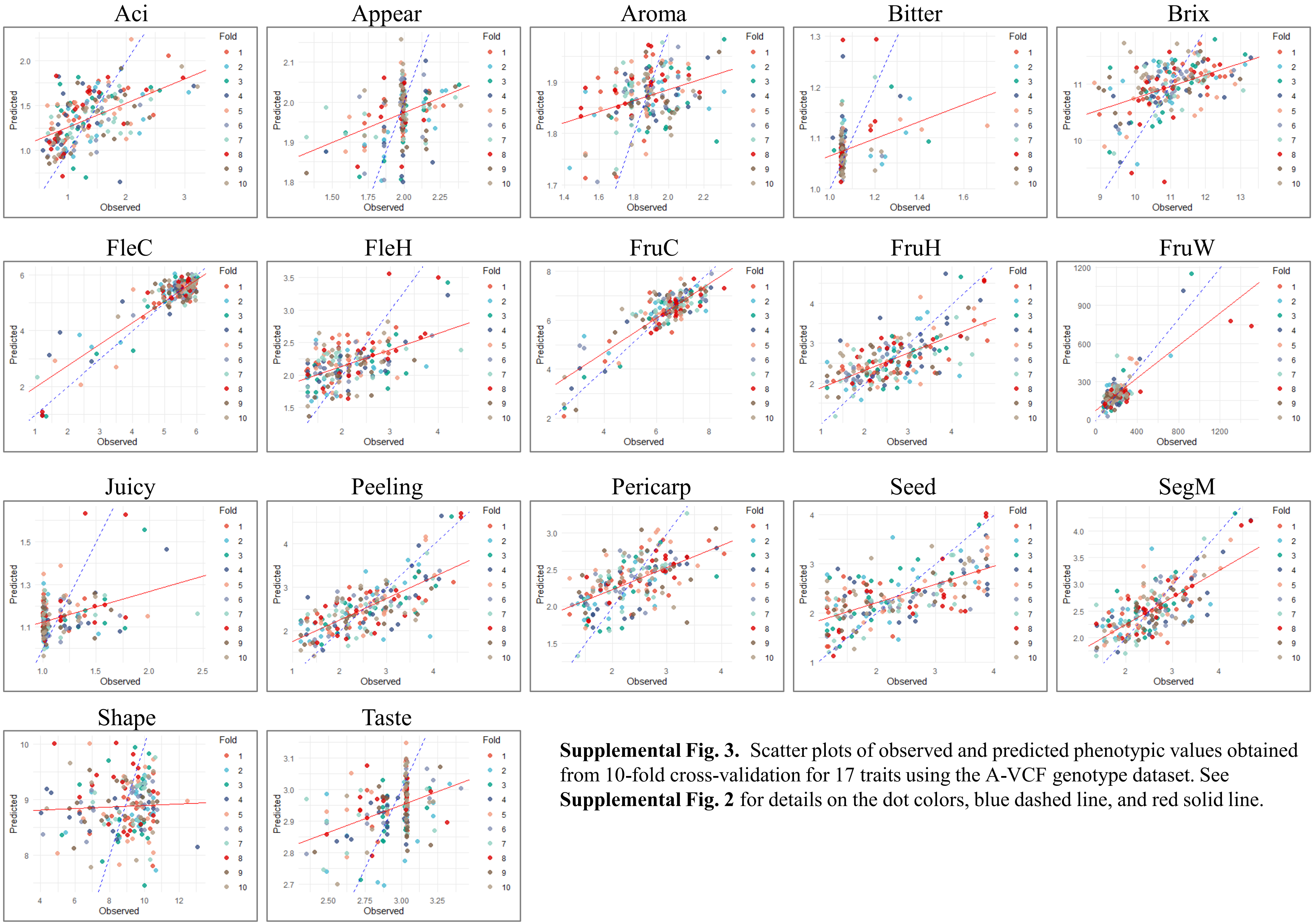

Supplemental Fig. 3. Scatter plots of observed and predicted phenotypic values obtained from 10-fold cross-validation for 17 traits using the A-VCF genotype dataset. See **Supplemental Fig. 2** for details on the dot colors, blue dashed line, and red solid line.

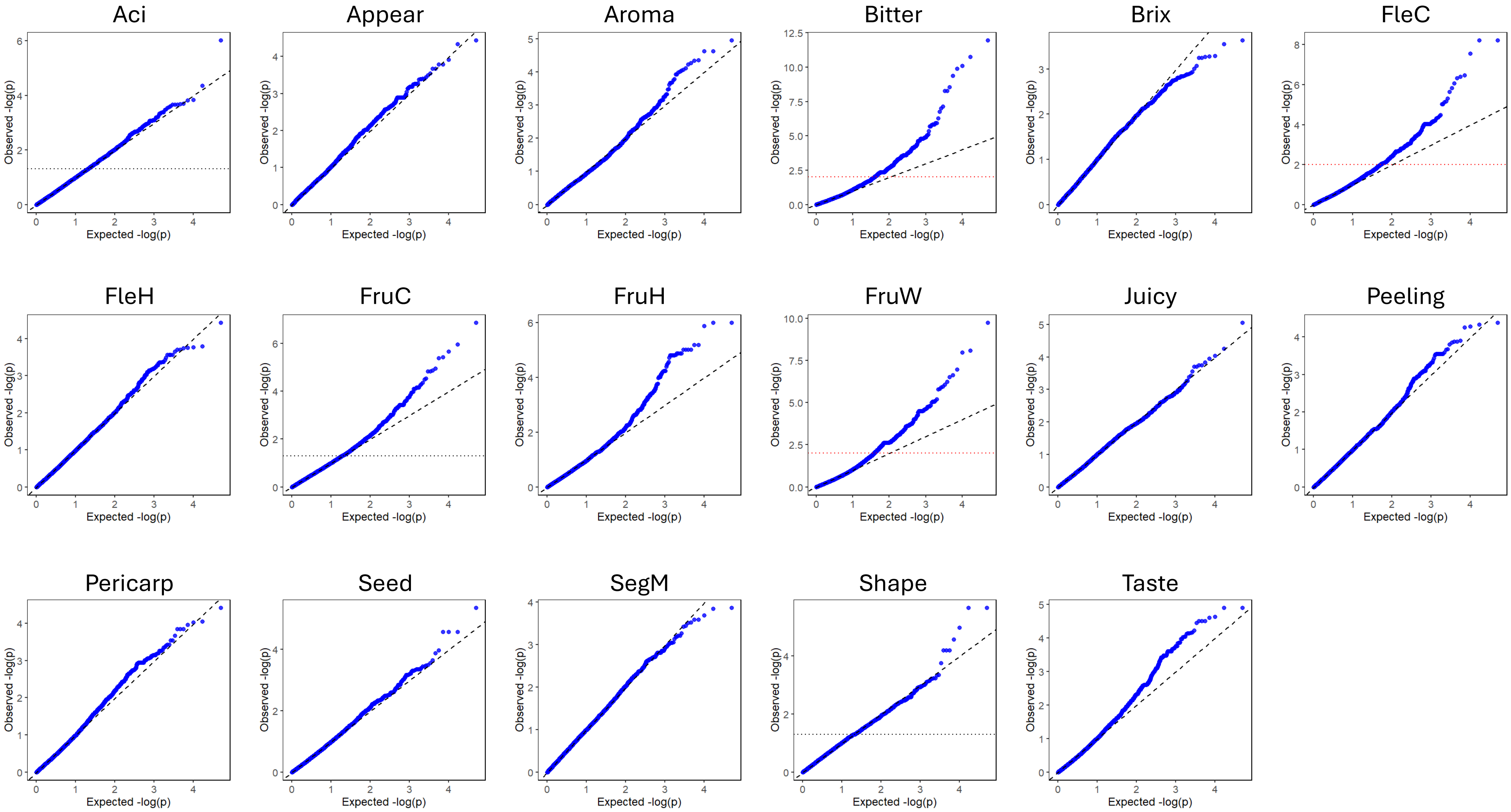

Supplemental Fig. 4. QQ plots of the observed and expected −log$_{10}$(p) values obtained from the GWAS analyses for 17 traits using the G02_2 iS-VCF genotype dataset. The diagonal black dashed line represents the null expectation under no association, and the horizontal dashed lines indicate the FDR thresholds (red for FDR = 0.01 and black for FDR = 0.05).

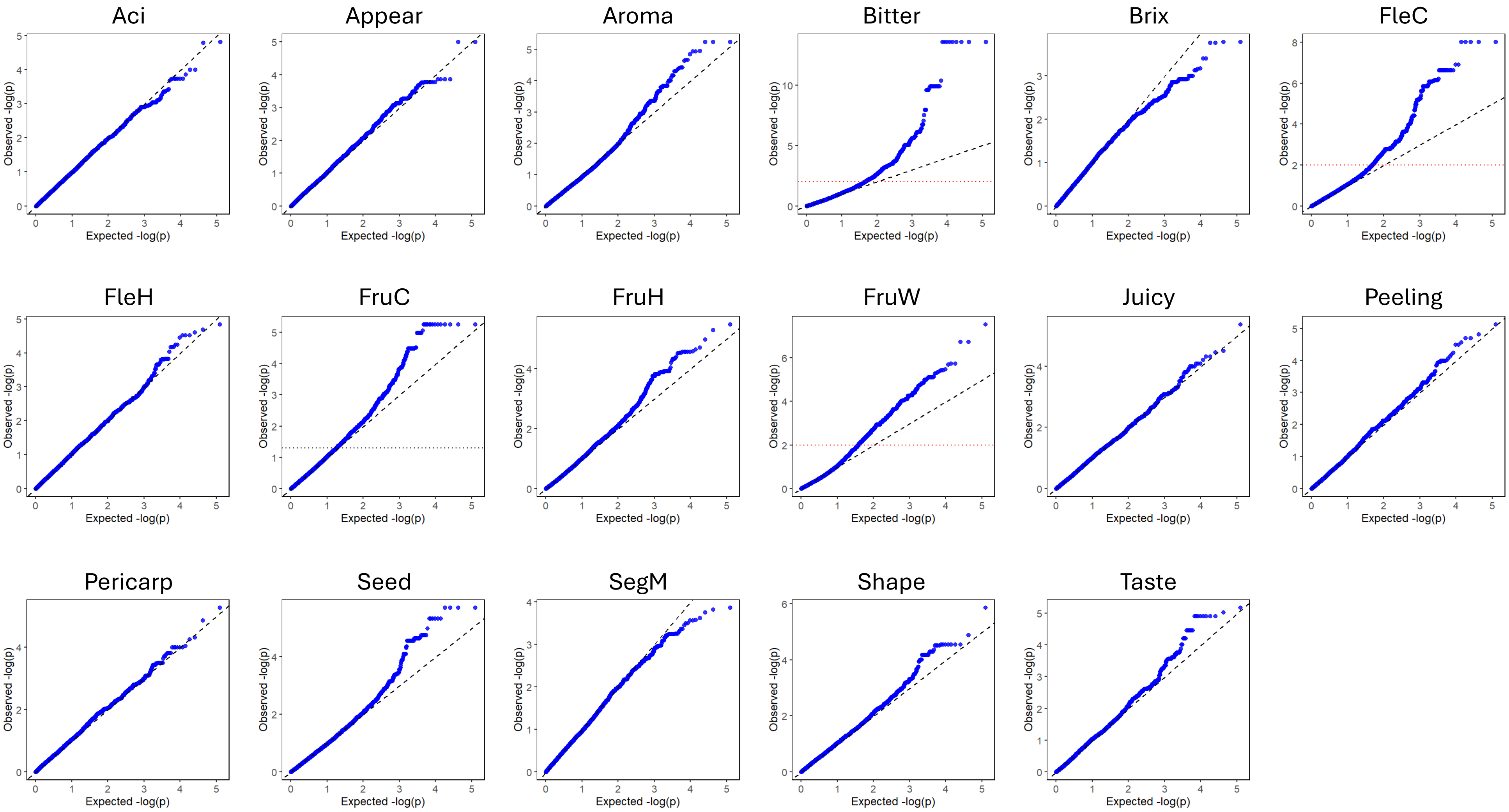

Supplemental Fig. 5. QQ plots for 17 traits using the G02_2 A-VCF genotype dataset. See **Supplemental Fig. 4** for details.
