# Supplementary material for: Facilitating genomic breeding in citrus via reference panel-based whole-genome imputation of low-coverage sequencing-derived genotype data
Source: Breed Sci. 2026 Feb 18;76(1):95–110. doi: 10.1270/jsbbs.25061 (PMC13437165; doi:10.1270/jsbbs.25061)
Supplement: Supplementary file 2 [file 76_095_s2.pdf]

doi: 10.1270/jsbbs.25061

Supplemental Table 1. List of the plant samples used for resequencing analysis

#	*Common name*	in R-VCF	Scientific name	Source	Category	Display name	SRA ID
1	Calamondin	O	*C. madurensis*	NIFTS	Variety	Calamondin	
2	Grapefruit Marsh	O	*C. paradisi*	NIFTS	Variety	Grapefruit_Marsh	
3	Hassaku	O	*C. hassaku*	NIFTS	Variety	Hassaku	
4	Hyuganatsu	O	*C. tamurana*	NIFTS	Variety	Hyuganatsu	
5	Kabosu	O	*C. sphaerocarpa*	NIFTS	Variety	Kabosu	
6	Kaikoukan	O	*C. truncata*	NIFTS	Variety	Kaikoukan	
7	Kawachi bankan	O	*C.* sp	NIFTS	Variety	Kawachibankan	
8	Kawano natsudaidai	O	*C. natsudaidai*	NIFTS	Variety	Natudaidai_Kawano	
9	Keraji	O	*C. keraji*	NIFTS	Variety	Keraji	
10	Kunenbo-A	O	*C. nobilis* Lour. *var. kunep*	NIFTS	Variety	Kunenbo-A	
11	Miyauchi Iyo	O	*C. iyo*	NIFTS	Variety	IyoMiyauchi	
12	Ogonkan	O	*C.* sp	NIFTS	Variety	Ogonkan	
13	Otachibana		*C. otachibana*	NIFTS	Variety	Otachibana	
14	Purut (C. hystrix)	O	*C. hystrix*	NIFTS	Variety	Purut	
15	Sour orange	O	*C. aurantium*	NIFTS	Variety	Daidai	
16	Sweet orange Trovita	O	*C. sinensis*	NIFTS	Variety	SwOrange_Trovita	
17	Tachibana-A	O	*C. tachibana* (Makino) Tanaka	NIFTS	Variety	Tachibana-A	
18	Tachibana-B	O	*C. tachibana* (Makino) Tanaka	NIFTS	Variety	Tachibana-B	
19	Yuzu	O	*C. junos*	NIFTS	Variety	Yuzu	
20	S_01	O	*C.* sp	NIFTS	Selection	2700OIy-25	
21	S_02	O	*C.* sp	NIFTS	Selection	62monoG30	
22	S_03	O	*C.* sp	NIFTS	Selection	AE0458	
23	S_04	O	*C.* sp	NIFTS	Selection	AF0243	
24	S_05	O	*C.* sp	NIFTS	Selection	E647	
25	S_06	O	*C.* sp	NIFTS	Selection	EnOw21	
26	S_07	O	*C.* sp	NIFTS	Selection	F060336	
27	S_08	O	*C.* sp	NIFTS	Selection	F090412	
28	S_09	O	*C.* sp	NIFTS	Selection	G1259	
29	S_10	O	*C.* sp	NIFTS	Selection	G-1267	
30	S_11	O	*C.* sp	NIFTS	Selection	K38	
31	S_12	O	*C.* sp	NIFTS	Selection	K49	
32	S_13	O	*C.* sp	NIFTS	Selection	K52	
33	S_14	O	*C.* sp	NIFTS	Selection	K53	
34	S_15	O	*C.* sp	NIFTS	Selection	K55	
35	S_16	O	*C.* sp	NIFTS	Selection	K58	
36	S_17	O	*C.* sp	NIFTS	Selection	K36	
37	S_18	O	*C.* sp	NIFTS	Selection	K54	
38	S_19	O	*C.* sp	NIFTS	Selection	KyEn4	
39	S_20	O	*C.* sp	NIFTS	Selection	KyOw14	
40	S_21	O	*C.* sp	NIFTS	Selection	KyOw21	
41	S_22	O	*C.* sp	NIFTS	Selection	L071094	
42	S_23	O	*C.* sp	NIFTS	Selection	No1011	
43	S_24	O	*C.* sp	NIFTS	Selection	No1408	
44	S_25	O	*C.* sp	NIFTS	Selection	Nou5gou	
45	S_26	O	*C.* sp	NIFTS	Selection	Nou8	
46	S_27	O	*C.* sp	NIFTS	Selection	O45	
47	S_28	O	*C.* sp	NIFTS	Selection	O63	
48	S_29	O	*C.* sp	NIFTS	Selection	O67	
49	S_30	O	*C.* sp	NIFTS	Selection	O68	
50	S_31	O	*C.* sp	NIFTS	Selection	O69	
51	S_32	O	*C.* sp	NIFTS	Selection	O70	
52	S_33	O	*C.* sp	NIFTS	Selection	O46	
53	S_34	O	*C.* sp	NIFTS	Selection	O56	
54	S_35	O	*C.* sp	NIFTS	Selection	O64	
55	S_36	O	*C.* sp	NIFTS	Selection	S061068	
56	S_37	O	*C.* sp	NIFTS	Selection	S3-15	
57	S_38	O	*C.* sp	NIFTS	Selection	S4-34	
58	S_39	O	*C.* sp	NIFTS	Selection	S5-18	
59	S_40	O	*C.* sp	NIFTS	Selection	S8-38	
60	S_41	O	*C.* sp	NIFTS	Selection	S8-66	
61	S_42	O	*C.* sp	NIFTS	Selection	U22	
62	Banpeiyu	O	*C. maxima*	NIFTS	pummelo	Banpeiyu	
63	Bansei Sham Buntan	O	*C. maxima*	NIFTS	pummelo	Late_Siam_Pummelo	
64	Benimadoka	O	*C. maxima*	NIFTS	pummelo	Benimadoka	
65	Chandler Pummelo	O	*C. maxima*	NIFTS	pummelo	ChandlerPummelo	
66	Hirado Buntan	O	*C. maxima*	NIFTS	pummelo	Hirado_Buntan	
67	Mato Buntan	O	*C. maxima*	NIFTS	pummelo	Mato_Buntan	
68	Siamese acidless pummelo		*C. maxima*	NIFTS	pummelo	SiameseAcidless	
69	Tanigawa Buntan	O	*C. maxima*	NIFTS	pummelo	TanigawaBuntan	
70	Tosa buntan	O	*C. maxima*	NIFTS	pummelo	Tosa_Buntan	
71	White type pummelo	O	*C. maxima*	NIFTS	pummelo	Pummelo_white_type	
72	Clementine	O	*C. clementina*	NIFTS	Mandarin	Clementine	
73	Dancy	O	*C. tangerina*	NIFTS	Mandarin	Dancy	
74	King mandarin	O	*C. nobilis*	NIFTS	Mandarin	King_mandarin	
75	Kishu	O	*C. kinokuni*	NIFTS	Mandarin	Kishu	
76	Kobeni mikan	O	*C. erythrosa*	NIFTS	Mandarin	Kobeni_Mikan	
77	Mediterranean mandarin	O	*C. deliciosa*	NIFTS	Mandarin	Med_mandarin	
78	Kishu 'Mukaku Kishu'		*C. kinokuni*	NIFTS	Mandarin	MukakuKishu	
79	Ponkan 'Yoshida'	O	*C. reticulata*	NIFTS	Mandarin	Ponkan_Yoshida	

80	Satsuma 'Miyagawa Wase'	O	*C. unshiu*	NIFTS	Mandarin	Satsuma_Miyagawa	
81	Sunki	O	*C. sunki*	NIFTS	Mandarin	Sunki	
82	Tachibana-C	O	*C. tachibana*	NIFTS	Mandarin	Tachibana-C	
83	Willowleaf mandarin	O	*C. deliciosa*	NIFTS	Mandarin	Willowleaf_mandarin	
84	Konta		*F. crassifolia*	NIFTS	Kumquat	Konta	
85	Nagami kumquat	O	*F. margarita*	NIFTS	Kumquat	Nagami_Kumquat	
86	Nimpo kumquat	O	*F. crassifolia*	NIFTS	Kumquat	Nimpo_Kumquat	
87	All Spice	O	*C.* sp	NIFTS	Hybrid	AllSpice	
88	Amakusa	O	*C.* sp	NIFTS	Hybrid	Amakusa	
89	Ariake	O	*C.* sp	NIFTS	Hybrid	Ariake	
90	Asuki	O	*C.* sp	NIFTS	Hybrid	Asuki	
91	Asumi	O	*C.* sp	NIFTS	Hybrid	Asumi	
92	Awa orange	O	*C.* sp	NIFTS	Hybrid	Awa_Orange	
93	Benimadonna (EK28)	O	*C.* sp	NIFTS	Hybrid	Benimadonna	
94	Encore	O	*C.* sp	NIFTS	Hybrid	Encore	
95	Fortune	O	*C.* sp	NIFTS	Hybrid	Fortune	
96	Harehime	O	*C.* sp	NIFTS	Hybrid	Harehime	
97	Harumi	O	*C.* sp	NIFTS	Hybrid	Harumi	
98	Kanpei	O	*C.* sp	NIFTS	Hybrid	Kanpei	
99	Kara mandarin	O	*C.* sp	NIFTS	Hybrid	Kara	
100	Kiyomi	O	*C.* sp	NIFTS	Hybrid	Kiyomi	
101	Lee	O	*C.* sp	NIFTS	Hybrid	Lee	
102	Limequat	O	*Fortunella hybrid*	NIFTS	Hybrid	Limequat	
103	Mihaya	O	*C.* sp	NIFTS	Hybrid	Mihaya	
104	Minneola	O	*C.* sp	NIFTS	Hybrid	Minneola	
105	Murcott	O	*C.* sp	NIFTS	Hybrid	Murcott	
106	Nanko	O	*C.* sp	NIFTS	Hybrid	Nanko	
107	Natsumi		*C.* sp	NIFTS	Hybrid	Natsumi	
108	Nishinokaori	O	*C.* sp	NIFTS	Hybrid	Nishinokaori	
109	Nou6gou	O	*C.* sp	NIFTS	Hybrid	Nou6gou	
110	Orangequat	O	*Fortunella hybrid*	NIFTS	Hybrid	Orangequat	
111	Orland	O	*C.* sp	NIFTS	Hybrid	Orland	
112	Osceola	O	*C.* sp	NIFTS	Hybrid	Osceola	
113	Page	O	*C.* sp	NIFTS	Hybrid	Page	
114	Rinoka	O	*C.* sp	NIFTS	Hybrid	Rinoka	
115	Robinson	O	*C.* sp	NIFTS	Hybrid	Robinson	
116	Seinanno hikari	O	*C.* sp	NIFTS	Hybrid	SeinannoHikari	
117	Seminole	O	*C.* sp	NIFTS	Hybrid	Seminole	
118	Setoka	O	*C.* sp	NIFTS	Hybrid	Setoka	
119	Shiranuhi	O	*C.* sp	NIFTS	Hybrid	Shiranuhi	
120	Southern Yellow	O	*C.* sp	NIFTS	Hybrid	Southern_Yellow	
121	Sweet Spring	O	*C.* sp	NIFTS	Hybrid	Sweetspring	
122	Tamami	O	*C.* sp	NIFTS	Hybrid	Tamami	
123	Tsunokagayaki	O	*C.* sp	NIFTS	Hybrid	Tsunokagayaki	
124	Tsunonozomi	O	*C.* sp	NIFTS	Hybrid	Tsunonozomi	
125	Wilking	O	*C.* sp	NIFTS	Hybrid	Wilking	
126	Youkou	O	*C.* sp	NIFTS	Hybrid	Youkou	
127	Akakunibu 1	O	*C. depressa*	SRA	Variety	Akakunibu1	SRR14458764
128	Akakunibu 2	O	*C. depressa*	SRA	Variety	Akakunibu2	SRR14458763
129	Calamondin		*C. madurensis*	SRA	Variety	Calamondin	SRR6188463
130	Clementine		*C. clementina*	SRA	Variety	Clementine	SRR6415870
131	Cleopatra		*C. reshni*	SRA	Variety	Cleopatra	SRR5128221
132	Cleopatra	O	*C. reshni*	SRA	Variety	Cleopatra_2	SRR14453878
133	Daidai	O	*C. aurantium*	SRA	Variety	Daidai	SRR14460014
134	Deedee	O	*C. luteo-turgida*	SRA	Variety	Deedee	SRR14509908
135	Grapefruit 'Duncan'		*C. paradisi*	SRA	Variety	Grapefruit_Duncan	SRR5128227
136	Grapefruit 'Flame'		*C. paradisi*	SRA	Variety	Grapefruit_Flame	SRR3927405
137	Grapefruit 'Marsh'		*C. paradisi*	SRA	Variety	Grapefruit_Marsh	SRR6188447
138	Grapefruit 'Ruby'		*C. paradisi*	SRA	Variety	Grapefruit_Ruby	SRR3927447
139	Grapefruit 'Triumph'		*C. paradisi*	SRA	Variety	Grapefruit_Triumph	SRR5128217
140	Indian Wild orange	O	*C. indica*	SRA	Variety	IndianWildOrange	SRR3948493
141	Izumi-kuganii	O	*C. depressa*	SRA	Variety	Izumi-kuganii	SRR14458770
142	Kaabishii shiikuwasha		*C. depressa*	SRA	Variety	Kaabishii_shiikuwasha	SRR14458787
143	Kaachii shiikuwasha	O	*C. depressa*	SRA	Variety	Kaachii_shiikuwasha	SRR14458775
144	Kabuchi	O	*C. kabuchii*	SRA	Variety	Kabuchi	SRR14509916
145	Katsuyama-Ishikunibu	O	*C. depressa*	SRA	Variety	Katsuyama-Ishikunibu	SRR14453874
146	Katsuyama-kuganii		*C. depressa*	SRA	Variety	Katsuyama-kuganii	SRR14458761
147	Keraji-mikan		*C. keraji*	SRA	Variety	Keraji-mikan	SRR14509911
148	King mandarin		*C. nobilis*	SRA	Variety	King_mandarin	SRR6188438
149	Kuganii shiikuwasha		*C. depressa*	SRA	Variety	Kuganii_shiikuwasha	SRR14458786
150	Kumquat 1	O	*F. sp.*	SRA	Variety	Kumquat_1	SRR6415868
151	Kunenbo 1	O	*C. nobilis* Lour. *var. kunep*	SRA	Variety	Kunenbo1	SRR14453877
152	Kunenbo 2	O	*C. nobilis* Lour. *var. kunep*	SRA	Variety	Kunenbo2	SRR14453876
153	Kunenbo 3		*C. nobilis* Lour. *var. kunep*	SRA	Variety	Kunenbo3	SRR14453875
154	Njakuganii shiikuwasha	O	*C. depressa*	SRA	Variety	Njakuganii_shiikuwasha	SRR14458765
155	Obeni		*C. tangerina*	SRA	Variety	Obeni_mikan	SRR14453873
156	Oogimi-Ishikuibu 1	O	*C. depressa*	SRA	Variety	Oogimi-Ishikunibu1	SRR13261962
157	Oogimi-Ishikunibu 2	O	*C. depressa*	SRA	Variety	Oogimi-Ishikunibu2	SRR14482356
158	Oogimi-Ishikunibu 3	O	*C. depressa*	SRA	Variety	Oogimi-Ishikunibu3	SRR14482355
159	Oogimi-Ishikunibu 4	O	*C. depressa*	SRA	Variety	Oogimi-Ishikunibu4	SRR14482354
160	Oogimi-kuganii shiikuwasha		*C. depressa*	SRA	Variety	Oogimi-kuganii_shiikuwasha	SRR14458762
161	Oto	O	*C. sinoglandis*	SRA	Variety	Oto	SRR14509917

162	Rokugatsu-mikan	O	*C. rokugatsu*	SRA	Variety	Rokugatsu-mikan	SRR14509909
163	Seedless-Shiikuwasha	O	*C. depressa*	SRA	Variety	Seedless-Shiikuwasha	SRR14458766
164	Shiikuwasha (Awadake) 1	O	*C. depressa*	SRA	Variety	Shiikuwasha_Awadake_1	SRR14458776
165	Shiikuwasha (Awadake) 4		*C. depressa*	SRA	Variety	Shiikuwasha_Awadake_4	SRR14458774
166	Shiikuwasha (Florida)		*C. depressa*	SRA	Variety	Shiikuwasha_Florida	SRR14458785
167	Shiikuwasha (Genga)		*C. depressa*	SRA	Variety	Shiikuwasha_Genga	SRR14458772
168	Shiikuwasha (Ginama) 9		*C. depressa*	SRA	Variety	Shiikuwasha_Ginama_9	SRR14458773
169	Shiikuwasha (Hiji)		*C. depressa*	SRA	Variety	Shiikuwasha_Hiji	SRR14458779
170	Shiikuwasha (Imadomari)	O	*C. depressa*	SRA	Variety	Shiikuwasha_Imadomari	SRR14458771
171	Shiikuwasha (Kaneshi)	O	*C. depressa*	SRA	Variety	Shiikuwasha29	SRR14509910
172	Shiikuwasha (Katsuudake) 1	O	*C. depressa*	SRA	Variety	Shiikuwasha_Katsuudake_1	SRR14458769
173	Shiikuwasha (Katsuudake) 5		*C. depressa*	SRA	Variety	Shiikuwasha_Katsuudake_5	SRR14458768
174	Shiikuwasha (Katsuudake) 6		*C. depressa*	SRA	Variety	Shiikuwasha_Katsuudake_6	SRR14458767
175	Shiikuwasha (Katsuyama)	O	*C. depressa*	SRA	Variety	Shiikuwasha_Katsuyama	SRR14458760
176	Shiikuwasha (Oku)		*C. depressa*	SRA	Variety	Shiikuwasha_Oku	SRR14458780
177	Shiikuwasha (Oogimi) 1	O	*C. depressa*	SRA	Variety	Shiikuwasha_Oogimi_1	SRR14458784
178	Shiikuwasha (Oogimi) 2	O	*C. depressa*	SRA	Variety	Shiikuwasha_Oogimi_2	SRR14458783
179	Shiikuwasha (Oogimi) 3		*C. depressa*	SRA	Variety	Shiikuwasha_Oogimi_3	SRR14458782
180	Shiikuwasha (Oogimi) 4		*C. depressa*	SRA	Variety	Shiikuwasha_Oogimi_4	SRR14458781
181	Shiikuwasha (Oshikawa) 1		*C. depressa*	SRA	Variety	Shiikuwasha_Oshikawa_1	SRR14458778
182	Shiikuwasha (Oshikawa) 2		*C. depressa*	SRA	Variety	Shiikuwasha_Oshikawa_2	SRR14458777
183	Shikinari-mikan		*C.* sp	SRA	Variety	Shikinari-mikan	SRR14460013
184	Tankan 1	O	*C. tankan*	SRA	Variety	Tankan1	SRR14453880
185	Tankan 2		*C. tankan*	SRA	Variety	Tankan2	SRR14453879
186	Tarogayo	O	*C.* sp	SRA	Variety	Tarogayo	SRR14509915
187	Unzoki	O	*C.* sp	SRA	Variety	Unzoki	SRR14509913
188	Yama Mikanguwa	O	*C. depressa*	SRA	Variety	Yama_Mikanguwa	SRR14458759
189	Yukunibu (Hama)		*C. yanbaruensis*	SRA	Variety	Yukunibu1	SRR14509914
190	Yukunibu (Ura)	O	*C. yanbaruensis*	SRA	Variety	Yukunibu2	SRR14509912
191	Yuzu (Nikou)		*C. junos*	SRA	Variety	Yuzu_Nikou	SRR14460018
192	Yuzu (Shingu)		*C. junos*	SRA	Variety	Yuzu_Shingu	SRR14460017
193	Cariappa pummelo	O	*C. maxima*	SRA	pummelo	Cariappa_pummelo	SRR5128235
194	Hunnan pummelo	O	*C. maxima*	SRA	pummelo	Hunnan_pummelo	SRR5128240
195	Kao Phuang pummelo	O	*C. maxima*	SRA	pummelo	Kao_Phuang_pummelo	SRR5128236
196	mandarin QH117	O	*C. maxima*	SRA	pummelo	mandarin_QH117	SRR3822244
197	Mato Buntan		*C. maxima*	SRA	pummelo	Mato_buntan	SRR5128244
198	Pummelo 10Z	O	*C. maxima*	SRA	pummelo	pummelo_10Z	SRR3823645
199	Pummelo 2011-GL-01	O	*C. maxima*	SRA	pummelo	pummelo_2011-GL-01	SRR3858312
200	Pummelo 28H	O	*C. maxima*	SRA	pummelo	pummelo_28H	SRR3823225
201	Pummelo GBY	O	*C. maxima*	SRA	pummelo	pummelo_GBY	SRR3823447
202	Pummelo GL-01	O	*C. maxima*	SRA	pummelo	pummelo_GL-01	SRR3822291
203	Pummelo HNHY	O	*C. maxima*	SRA	pummelo	pummelo_HNHY	SRR3823230
204	Pummelo HWB5 3	O	*C. maxima*	SRA	pummelo	pummelo_HWB5_3	SRR4294658
205	Pummelo Q-04	O	*C. maxima*	SRA	pummelo	pummelo_Q-04	SRR3823455
206	Pummelo RL-06	O	*C. maxima*	SRA	pummelo	pummelo_RL-06	SRR3823409
207	Pummelo SR3-2	O	*C. maxima*	SRA	pummelo	pummelo_SR3-2	SRR3822290
208	Pummelo SYBS	O	*C. maxima*	SRA	pummelo	pummelo_SYBS	SRR3844987
209	Pummelo SYPS	O	*C. maxima*	SRA	pummelo	pummelo_SYPS	SRR3824065
210	Pummelo WBY	O	*C. maxima*	SRA	pummelo	pummelo_WBY	SRR3823251
211	Pummelo WSY	O	*C. maxima*	SRA	pummelo	pummelo_WSY	SRR5796633
212	Pummelo YNMD	O	*C. maxima*	SRA	pummelo	pummelo_YNMD	SRR9127777
213	Pummelo YNSJ	O	*C. maxima*	SRA	pummelo	pummelo_YNSJ	SRR9127776
214	Pummelo ZGST	O	*C. maxima*	SRA	pummelo	pummelo_ZGST	SRR9127780
215	Tahitian pummelo	O	*C. maxima*	SRA	pummelo	Tahitian_pummelo	SRR5128237
216	Flying dragon		*P. trifoliata*	SRA	Poncirus	Karatachi_Flying_dragon_2	SRR12323703
217	Karatachi Flying dragon	O	*P. trifoliata*	SRA	Poncirus	Karatachi_Flying_dragon	SRR5128234
218	Little leaf	O	*P. trifoliata*	SRA	Poncirus	Karatachi_Little_leaf	SRR12323705
219	Rubidoux	O	*P. trifoliata*	SRA	Poncirus	Karatachi_Rubidoux	SRR12323702
220	Trifoliate orange	O	*C. trifoliata*	SRA	Poncirus	Trifoliate_orange	SRR6188465
221	Citrus hongheensis	O	*C. hongheensis*	SRA	Papeda	Honghe_papeda	SRR12494834
222	Ichang papeda		*C. cavaleriei*	SRA	Papeda	Ichang_papeda	SRR6188454
223	Ichang Papeda DYC	O	*C. ichangensis*	SRA	Papeda	IchangPapeda_DYC	SRR3929735
224	Ichang Papeda JF	O	*C. ichangensis*	SRA	Papeda	IchangPapeda_JF	SRR3929760
225	Ichang Papeda PY11	O	*C. ichangensis*	SRA	Papeda	IchangPapeda_PY11	SRR3928244
226	Ichang Papeda TK	O	*C. ichangensis*	SRA	Papeda	IchangPapeda_TK	SRR3929810
227	Ichang Papeda XC	O	*C. ichangensis*	SRA	Papeda	IchangPapeda_XC	SRR3929903
228	Ichang Papeda XJC	O	*C. ichangensis*	SRA	Papeda	IchangPapeda_XJC	SRR4006657
229	Ichang Papeda YCC	O	*C. ichangensis*	SRA	Papeda	IchangPapeda_YCC	SRR3928564
230	Ichang Papeda YCLS	O	*C. ichangensis*	SRA	Papeda	IchangPapeda_YCLS	SRR3929790
231	Ichang Papeda YCYJ	O	*C. ichangensis*	SRA	Papeda	IchangPapeda_YCYJ	SRR3929943
232	Ichang Papeda YL	O	*C. ichangensis*	SRA	Papeda	IchangPapeda_YL	SRR3930078
233	Ichang Papeda ZY	O	*C. ichangensis*	SRA	Papeda	IchangPapeda_ZY	SRR3931949
234	Micrantha Biasong	O	*C. micrantha*	SRA	Papeda	Micrantha_Biasong	SRR6188461
235	Yuanjiang wild Ichang papeda		*C. ichangensis*	SRA	Papeda	Yuanjiang_papeda	SRR12494594
236	Changsha mandarin	O	*C. reticulata*	SRA	Mandarin	Changsha_mandarin	SRR5128226
237	Changsha mandarin2		*C. reticulata*	SRA	Mandarin	Changsha_mandarin2	SRR6188440
238	Cleopatra mandarin		*C. reshni*	SRA	Mandarin	Cleopatra_mandarin	SRR6188441
239	Dancy tangerine1		*C. reticulata*	SRA	Mandarin	Dancy_tangerine1	SRR5128243
240	Dancy tangerine2		*C. tangerina*	SRA	Mandarin	Dancy_tangerine2	SRR6188439
241	Kishu mandarin	O	*C. kinokuni*	SRA	Mandarin	Kishu_mandarin	SRR6188456
242	KSH		*C. kinokuni*	SRA	Mandarin	KSH_Citrus_kinokuni	SRR6188456
243	Mandarin 18H	O	*C. reticulata*	SRA	Mandarin	mandarin_18H	SRR3749605

244	Mandarin 19P	O	*C. reticulata*	SRA	Mandarin	mandarin_19P	SRR3747617
245	Mandarin 20H	O	*C. reticulata*	SRA	Mandarin	mandarin_20H	SRR3747635
246	Mandarin 8L	O	*C. reticulata*	SRA	Mandarin	mandarin_8L	SRR3747335
247	Mandarin BTJ	O	*C. reticulata*	SRA	Mandarin	mandarin_BTJ	SRR3756893
248	Mandarin CDYN-24	O	*C. reticulata*	SRA	Mandarin	mandarin_CDYN-24	SRR5861493
249	Mandarin CSNJ	O	*C. reticulata*	SRA	Mandarin	mandarin_CSNJ	SRR3747609
250	Mandarin CYY	O	*C. reticulata*	SRA	Mandarin	mandarin_CYY	SRR3747399
251	Mandarin CZG		*C. reticulata*	SRA	Mandarin	mandarin_CZG	SRR3747583
252	Mandarin GD-137	O	*C. reticulata*	SRA	Mandarin	mandarin_GD-137	SRR5861469
253	Mandarin GDYN-81	O	*C. reticulata*	SRA	Mandarin	mandarin_GDYN-81	SRR5861519
254	Mandarin HFP1	O	*C. reticulata*	SRA	Mandarin	mandarin_HFP1	SRR4265832
255	Mandarin HFS1	O	*C. reticulata*	SRA	Mandarin	mandarin_HFS1	SRR4266018
256	Mandarin HPJ		*C. reticulata*	SRA	Mandarin	mandarin_HPJ	SRR3750611
257	Mandarin HZ	O	*C. reticulata*	SRA	Mandarin	mandarin_HZ	SRR3747527
258	Mandarin KYM	O	*C. reticulata*	SRA	Mandarin	mandarin_KYM	SRR3820643
259	Mandarin LHJ	O	*C. reticulata*	SRA	Mandarin	mandarin_LHJ	SRR10163368
260	Mandarin LRH		*C. reticulata*	SRA	Mandarin	mandarin_LRH	SRR10163367
261	Mandarin LYJ	O	*C. reticulata*	SRA	Mandarin	mandarin_LYJ	SRR3756887
262	Mandarin Mangshan	O	*C. reticulata*	SRA	Mandarin	mandarin_Mangshan	SRR5837537
263	Mandarin MLTJ	O	*C. reticulata*	SRA	Mandarin	mandarin_MLTJ	SRR3750679
264	Mandarin MSJ		*C. reticulata*	SRA	Mandarin	mandarin_MSJ	SRR3751832
265	Mandarin NFJ		*C. reticulata*	SRA	Mandarin	mandarin_NFJ	SRR5796630
266	Mandarin NJ	O	*C. reticulata*	SRA	Mandarin	mandarin_NJ	SRR3750668
267	Mandarin ORI		*C. reticulata*	SRA	Mandarin	mandarin_ORI	SRR3820595
268	Mandarin S6		*C. reticulata*	SRA	Mandarin	mandarin_S6	SRR10163366
269	Mandarin S8		*C. reticulata*	SRA	Mandarin	mandarin_S8	SRR10163365
270	Mandarin SJ		*C. reticulata*	SRA	Mandarin	mandarin_SJ	SRR3747529
271	Mandarin SPG	O	*C. reticulata*	SRA	Mandarin	mandarin_SPG	SRR3747540
272	Mandarin STJ	O	*C. reticulata*	SRA	Mandarin	mandarin_STJ	SRR3756933
273	Mandarin SW-374	O	*C. reticulata*	SRA	Mandarin	mandarin_SW-374	SRR5864746
274	Mandarin WLK	O	*C. reticulata*	SRA	Mandarin	mandarin_WLK	SRR3820551
275	Mandarin YSJ	O	*C. reticulata*	SRA	Mandarin	mandarin_YSJ	SRR3750648
276	MukakuKishu 2		*C. kinokuni*	SRA	Mandarin	MukakuKishu_2	SRR5128231
277	Satsuma		*C. unshiu*	SRA	Mandarin	Satsuma	SRR6188446
278	Sun Chu Sha Kat mandarin		*C. reticulata*	SRA	Mandarin	Sun_Chu_Sha_Kat_mandarin	SRR6188448
279	SunChuShaKat mandarin	O	*C. reticulata*	SRA	Mandarin	SunChuShaKat_mandarin	SRR5128232
280	Sunki		*C. sunki*	SRA	Mandarin	Sunki	SRR5128224
281	Sunki mandarin		*C. sunki*	SRA	Mandarin	Sunki_mandarin	SRR6188455
282	Tachibana (Arima) 1		*C. tachibana*	SRA	Mandarin	Tachibana_Arima_1	SRR14424830
283	Tachibana (Arima) 2		*C. tachibana*	SRA	Mandarin	Tachibana_Arima_2	SRR14424829
284	Tachibana (Hashimoto)		*C. tachibana*	SRA	Mandarin	Tachibana_Hashimoto	SRR14424833
285	Tachibana (Nikou) 1		*C. tachibana*	SRA	Mandarin	Tachibana_Nikou1	SRR14424828
286	Tachibana (Nikou) 2		*C. tachibana*	SRA	Mandarin	Tachibana_Nikou2	SRR14424827
287	Tachibana (Shingu)		*C. tachibana*	SRA	Mandarin	Tachibana_Shingu	SRR14424832
288	Tachibana (Suno)		*C. tachibana*	SRA	Mandarin	Tachibana_Suno	SRR14424831
289	Tachibana 1		*C. tachibana*	SRA	Mandarin	Tachibana_1	SRR5128228
290	Tachibana 2		*C. tachibana*	SRA	Mandarin	Tachibana_2	SRR6188457
291	Tanibuta (Ginama) 3	O	*C. reticulata*	SRA	Mandarin	Tanibuta_1	SRR14482353
292	Tanibuta (Ginama) 4	O	*C. reticulata*	SRA	Mandarin	Tanibuta_2	SRR14482352
293	Tanibuta (Isagawa)	O	*C. reticulata*	SRA	Mandarin	Tanibuta_4	SRR12854171
294	Tanibuta (Oshikawa) 4	O	*C. reticulata*	SRA	Mandarin	Tanibuta_3	SRR14482351
295	TBM	O	*C. tachibana*	SRA	Mandarin	TBM_Citrus_tachibana	SRR6188457
296	Lemon Eureka		*C. limon*	SRA	Lemon_Lime	Lemon_Eureka	SRR6188464
297	Lisbon lemon	O	*C. limon*	SRA	Lemon_Lime	Lisbon_lemon	SRR5128241
298	Mexican lime 1	O	*C. aurantiifolia*	SRA	Lemon_Lime	Mexican_lime_1	SRR5128245
299	Mexican lime 2	O	*C. aurantiifolia*	SRA	Lemon_Lime	Mexican_lime_2	SRR6188466
300	Rangpur lime	O	*C. limonia*	SRA	Lemon_Lime	Rangpur_lime	SRR6188467
301	Red rough lemon	O	*C. jambhiri*	SRA	Lemon_Lime	Red_rough_lemon	SRR6188444
302	Schaub rough lemon	O	*C. jambhiri*	SRA	Lemon_Lime	Schaub_rough_lemon	SRR5128225
303	kumquat	O	*C. hindsii*	SRA	Kumquat	Kumquat_2	SRR8983184
304	Kumquat margarita	O	*F. margarita*	SRA	Kumquat	Kumquat_margarita	SRR5128233
305	Carrizo citrange	O	*C.* sp	SRA	Hybrid	Carrizo_citrange	SRR12323700
306	Fairchild	O	*C.* sp	SRA	Hybrid	Fairchild	SRR5154632
307	GFxTO 20-3	O	*C.* sp	SRA	Hybrid	GFxTO_20-3	SRR1209974
308	Pummelo x Citron	O	*C.* sp	SRA	Hybrid	PummeloxCitron	SRR14460010
309	SRA GFxTO 16-10	O	*C.* sp	SRA	Hybrid	GFxTO_16-10	SRR1209583
310	Swingle citrumelo	O	*C. paradisi* x *C. trifoliata*	SRA	Hybrid	Swingle_citrumelo	SRR1033838
311	Buddhas hand citron 2	O	*C. medica*	SRA	Citron	Buddhas_hand_citron_2	SRR6188453
312	Bushukan 1	O	*C. medica*	SRA	Citron	Bushukan1	SRR14460012
313	Bushukan 2		*C. medica*	SRA	Citron	Bushukan2	SRR14460011
314	Citron JY	O	*C. medica*	SRA	Citron	Citron_JY	SRR3938734
315	Citron JY8	O	*C. medica*	SRA	Citron	Citron_JY8	SRR3944160
316	citron XZ	O	*C. medica*	SRA	Citron	citron_XZ	SRR4010249
317	Citron XZ1	O	*C. medica*	SRA	Citron	Citron_XZ1	SRR3938253
318	Corsica citron	O	*C. medica*	SRA	Citron	Corsica_citron	SRR6188458
319	Ethrog citron	O	*C. medica*	SRA	Citron	Ethrog_citron	SRR5128222
320	Humpang citron	O	*C. medica*	SRA	Citron	Humpang_citron	SRR6188451
321	Mac Veu Mountain citron	O	*C. medica*	SRA	Citron	Mac_Veu_Mountain_citron	SRR6188459
322	Persistent Stigma citron	O	*C. medica*	SRA	Citron	Persistent_Stigma_citron	SRR5128216

Supplemental Table 2. List of the plant samples used for GRAS-Di genotyping study

#	Common name	Category
1	Grapefruit 'Marsh'	Variety
2	Hassaku	Variety
3	Iyo 'Miyauchi'	Variety
4	Kawachi Bankan	Variety
5	King mandarin	Variety
6	Natsudaidai 'Kawano'	Variety
7	Ponkan 'Yoshida'	Variety
8	Satsuma 'Miyagawa wase'	Variety
9	Soren Tangelo	Variety
10	Sweet orange 'Trovita'	Variety
11	Tankan 'Tarumizu 1 gou'	Variety
12	Yuge Hyoukan	Variety
13	Clementine	Mandarin
14	Dancy	Mandarin
15	Hyuganatsu	Mandarin
16	Kishu 'Mukaku Kishu'	Mandarin
17	Mediterranean Mandarin	Mandarin
18	Banpeiyu	pummelo
19	Egami Buntan	pummelo
20	Hirado Buntan	pummelo
21	Mato Buntan	pummelo
22	Akemi	Hybrid
23	Aki tangol	Hybrid
24	All Spice	Hybrid
25	Amaka	Hybrid
26	Amakusa	Hybrid
27	Ariake	Hybrid
28	Asumi	Hybrid
29	Benibae	Hybrid
30	Ehime Kashi No.28	Hybrid
31	Encore	Hybrid
32	Fairchild	Hybrid
33	Harehime	Hybrid
34	Hareyaka	Hybrid
35	Haruhi	Hybrid
36	Haruka	Hybrid
37	Harumi	Hybrid
38	Hayaka	Hybrid
39	Kanpei	Hybrid
40	Kara	Hybrid
41	Kiyomi	Hybrid
42	Lee	Hybrid
43	Mihaya	Hybrid
44	Mihocore	Hybrid
45	Murcott	Hybrid
46	Nanko	Hybrid
47	Nishinokaori	Hybrid
48	Nou 5 gou	Hybrid
49	Nou 6 gou	Hybrid
50	Nova	Hybrid
51	Osceolla	Hybrid
52	Page	Hybrid
53	Reikou	Hybrid
54	Robinson	Hybrid
55	Seiho	Hybrid
56	Seinannohikari	Hybrid
57	Seminole	Hybrid
58	Setoka	Hybrid
59	Setomi	Hybrid
60	Shiranuhi	Hybrid
61	Southern Red	Hybrid
62	Southern Yellow	Hybrid

63	Sweet Spring	Hybrid
64	Tamami	Hybrid
65	Tsunokagayaki	Hybrid
66	Tsunokaori	Hybrid
67	Tsunonozomi	Hybrid
68	Wilking	Hybrid
69	Yoko	Hybrid
70	S_33	Selection
71	S_43	Selection
72	S_44	Selection
73	S_45	Selection
74	S_23	Selection
75	S_24	Selection
76	S_06	Selection
77	S_19	Selection
78	S_46	Selection
79	S_20	Selection
80	S_21	Selection
81	S_34	Selection
82	S_42	Selection
83	S_47	Selection
84	S_05	Selection
85	S_48	Selection
86	S_22	Selection
87	S_49	Selection
88	S_50	Selection
89	S_51	Selection
90	S_52	Selection
91	S_53	Selection
92	S_54	Selection
93	S_55	Selection
94	S_28	Selection
95	S_29	Selection
96	S_56	Selection
97	Line_01-01	Selection
98	Line_01-02	Selection
99	Line_01-03	Selection
100	Line_01-04	Selection
101	Line_01-05	Selection
102	Line_01-06	Selection
103	Line_01-07	Selection
104	Line_01-08	Selection
105	Line_01-09	Selection
106	Line_01-10	Selection
107	Line_01-11	Selection
108	Line_01-12	Selection
109	Line_01-13	Selection
110	Line_01-14	Selection
111	Line_01-15	Selection
112	Line_01-16	Selection
113	Line_01-17	Selection
114	Line_01-18	Selection
115	Line_01-19	Selection
116	Line_01-20	Selection
117	Line_01-21	Selection
118	Line_01-22	Selection
119	Line_01-23	Selection
120	Line_01-24	Selection
121	Line_02-01	Selection
122	Line_02-02	Selection
123	Line_02-03	Selection
124	Line_02-04	Selection
125	Line_02-05	Selection
126	Line_02-06	Selection
127	Line_02-07	Selection

128	Line_02-08	Selection
129	Line_02-09	Selection
130	Line_02-10	Selection
131	Line_02-11	Selection
132	Line_02-12	Selection
133	Line_02-13	Selection
134	Line_02-14	Selection
135	Line_02-15	Selection
136	Line_02-16	Selection
137	Line_02-17	Selection
138	Line_02-18	Selection
139	Line_03-01	Selection
140	Line_03-02	Selection
141	Line_03-03	Selection
142	Line_03-04	Selection
143	Line_03-05	Selection
144	Line_03-06	Selection
145	Line_03-07	Selection
146	Line_03-08	Selection
147	Line_03-09	Selection
148	Line_03-10	Selection
149	Line_03-11	Selection
150	Line_03-12	Selection
151	Line_03-13	Selection
152	Line_03-14	Selection
153	Line_03-15	Selection
154	Line_03-16	Selection
155	Line_03-17	Selection
156	Line_03-18	Selection
157	Line_03-19	Selection
158	Line_03-20	Selection
159	Line_03-21	Selection
160	Line_03-22	Selection
161	Line_03-23	Selection
162	Line_03-24	Selection
163	Line_03-25	Selection
164	Line_03-26	Selection
165	Line_04-01	Selection
166	Line_04-02	Selection
167	Line_04-03	Selection
168	Line_04-04	Selection
169	Line_04-05	Selection
170	Line_04-06	Selection
171	Line_04-07	Selection
172	Line_04-08	Selection
173	Line_04-09	Selection
174	Line_04-10	Selection
175	Line_04-11	Selection
176	Line_04-12	Selection
177	Line_04-13	Selection
178	Line_04-14	Selection
179	Line_04-15	Selection
180	Line_04-16	Selection
181	Line_04-17	Selection
182	Line_04-18	Selection
183	Line_04-19	Selection
184	Line_04-20	Selection
185	Line_05-01	Selection
186	Line_05-02	Selection
187	Line_05-03	Selection
188	Line_05-04	Selection
189	Line_05-05	Selection
190	Line_05-06	Selection
191	Line_05-07	Selection
192	Line_06	Selection

Supplemental Table 3 Whole-genome imputation accuracies evaluated with 20 shared samples by R-VCF and S-VCF

A. A summary table of the imputation accuracy scores for 8 dataset evaluated with 20 common cultivars

Cultivars		G07_1	G07_2	G05_1	G05_2	G02_1	G02_2	G01_1	G01_2			average	Stdev
	Dataset	G07_1	G07_2	G05_1	G05_2	G02_1	G02_2	G01_1	G01_2				
	minMQ	39	40	39	40	39	40	39	40				
	Loci in A-VCF	85,603	85,877	82,989	83,095	72,597	72,869	66,042	65,707				
Clementine		75.1%	76.0%	75.0%	75.9%	77.8%	78.0%	78.5%	78.2%			76.8%	1.46%
Banpeiyu		75.6%	75.8%	75.6%	76.3%	78.0%	76.7%	78.0%	78.6%			76.8%	1.19%
Hirado buntan		75.7%	75.9%	75.8%	77.3%	78.2%	77.8%	78.1%	78.5%			77.2%	1.18%
Mato buntan		75.2%	74.7%	74.6%	76.3%	77.4%	76.7%	77.3%	78.1%			76.3%	1.31%
Satsuma		72.5%	72.9%	73.4%	74.4%	75.6%	74.4%	75.4%	75.5%			74.3%	1.20%
Sweet orange		65.0%	65.0%	65.8%	66.8%	67.7%	68.4%	69.4%	68.4%			67.1%	1.69%
Ponkan		78.5%	78.5%	78.4%	79.4%	80.6%	80.3%	80.8%	80.8%			79.7%	1.09%
Hassaku		62.5%	61.9%	63.7%	63.4%	67.6%	67.1%	67.7%	67.9%			65.2%	2.58%
Natsudaidai		61.6%	60.6%	62.9%	62.5%	65.5%	65.9%	66.4%	67.9%			64.2%	2.60%
Grapefruit		65.6%	65.1%	65.9%	66.8%	68.3%	67.2%	67.3%	68.1%			66.8%	1.16%
Iyo		68.0%	67.8%	69.0%	68.9%	70.5%	71.1%	73.0%	72.6%			70.1%	2.00%
Dancy		80.8%	80.4%	80.1%	81.5%	82.0%	82.1%	81.9%	82.0%			81.4%	0.79%
Hyuganatsu		59.6%	59.0%	60.9%	61.2%	62.5%	61.7%	62.4%	63.2%			61.3%	1.43%
Kishu		78.4%	78.2%	78.7%	79.6%	80.3%	79.3%	80.4%	80.2%			79.4%	0.88%
Kawachi bankan		63.2%	63.7%	65.2%	65.5%	66.7%	67.2%	67.8%	68.6%			66.0%	1.93%
Kiyomi		68.2%	67.7%	68.8%	69.1%	71.0%	70.6%	71.6%	71.5%			69.8%	1.54%
Encore		77.6%	77.5%	77.3%	78.7%	78.4%	78.7%	78.0%	78.7%			78.1%	0.56%
Murcot		77.0%	76.3%	76.9%	77.8%	78.9%	78.8%	78.1%	79.1%			77.9%	1.03%
Osceola		78.3%	78.5%	77.9%	78.7%	78.9%	78.4%	79.0%	78.4%			78.5%	0.36%
Wilking		83.0%	83.2%	83.1%	84.1%	83.8%	83.7%	83.7%	84.2%			83.6%	0.47%
Average		72.1%	71.9%	72.5%	73.2%	74.5%	74.2%	74.7%	75.0%	75.0%	max	83.6%	2.60%
Stdev		7.15%	7.34%	6.57%	6.98%	6.32%	6.26%	6.07%	5.95%	71.9%	min	61.3%	0.36%

B. Mean of imputation accuracy scores for minGQ selection threshold

Group	average	Stdev
MQ<39 group	73.4%	1.37%
MQ<40 group	73.6%	1.33%

C. Heterozygosity score of the evaluated samples in R-VCF

Cultivars		G07_1	G07_2	G05_1	G05_2	G02_1	G02_2	G01_1	G01_2			average	Stdev
	Dataset	G07_1	G07_2	G05_1	G05_2	G02_1	G02_2	G01_1	G01_2				
	minMQ	39	40	39	40	39	40	39	40				
	Loci in A-VCF	85,603	85,877	82,989	83,095	72,597	72,869	66,042	65,707				
Clementine		44.6%	44.7%	45.2%	45.1%	45.4%	45.8%	45.1%	44.9%			45.1%	0.37%
Banpeiyu		7.19%	7.13%	7.13%	6.92%	6.09%	6.29%	5.87%	6.13%			6.6%	0.55%
Hirado buntan		7.55%	7.46%	7.60%	7.12%	6.50%	6.49%	6.27%	6.36%			6.9%	0.57%
Mato buntan		7.41%	7.37%	7.41%	7.08%	6.06%	6.31%	5.88%	5.94%			6.7%	0.70%
Satsuma		44.7%	45.0%	45.3%	44.8%	44.3%	44.9%	45.1%	44.6%			44.8%	0.31%
Sweet orange		73.0%	73.7%	73.4%	73.2%	73.6%	73.9%	73.2%	73.5%			73.4%	0.30%
Ponkan		29.5%	29.9%	30.1%	29.8%	29.5%	29.5%	29.8%	29.7%			29.7%	0.20%
Hassaku		70.8%	70.6%	71.2%	71.3%	72.3%	72.0%	73.5%	73.3%			71.9%	1.08%
Natsudaidai		81.7%	81.8%	82.3%	82.0%	83.3%	83.3%	83.6%	84.0%			82.7%	0.90%
Grapefruit		62.7%	62.4%	62.9%	62.6%	63.3%	63.0%	64.1%	63.4%			63.1%	0.52%
Iyo		65.2%	65.2%	65.0%	65.2%	66.7%	66.4%	66.7%	66.6%			65.9%	0.78%
Dancy		24.7%	25.0%	25.2%	25.0%	25.2%	25.1%	25.2%	24.8%			25.0%	0.19%
Hyuganatsu		66.2%	66.7%	66.5%	66.4%	66.8%	66.8%	67.0%	67.0%			66.7%	0.29%
Kishu		21.0%	21.4%	21.2%	21.3%	21.0%	21.3%	20.7%	20.5%			21.1%	0.31%
Kawachi bankan		57.5%	57.8%	58.6%	58.3%	59.5%	59.0%	59.8%	60.3%			58.9%	0.98%
Kiyomi		61.6%	62.5%	62.4%	62.0%	62.5%	62.4%	62.4%	62.6%			62.3%	0.34%
Encore		30.4%	30.5%	31.2%	30.4%	30.8%	30.8%	31.5%	30.9%			30.8%	0.39%
Murcot		32.0%	32.3%	33.0%	32.3%	31.5%	31.6%	31.9%	31.0%			31.9%	0.60%
Osceola		34.1%	34.7%	34.6%	34.7%	34.7%	35.0%	34.9%	35.6%			34.8%	0.44%
Wilking		15.8%	16.1%	16.1%	15.9%	15.7%	16.2%	15.4%	15.8%			15.9%	0.25%
Average		41.9%	42.1%	42.3%	42.1%	42.2%	42.3%	42.4%	42.3%	42.4%	max	82.7%	1.08%
Stdev		24.10%	24.16%	24.18%	24.26%	24.87%	24.74%	25.03%	25.06%	41.9%	min	6.6%	0.19%

Supplemental Table 4 Sumary of functional annotations of A-VCF and iS-VCF deduced with SnpEff

A: A-VCF

		Number of effects by impact				By functional class			Region									
Data set	Loci	High	Low	Moderate	Modifier	Missense	Nonsense	Synonymous	DOWNSTREAM	EXON	INTERGENIC	INTRON	SPLICE_SITE ACCEPTOR	SPLICE_SITE DONOR	SPLICE_SITE REGION	UPSTREAM	UTR_3_PRIME	UTR_5_PRIME
G07_1	85,603	152	12,292	13,928	78,534	50,985	39,043	12,093	82,191	35,472	18,031	63,331	29	30	2,998	61,137	9,794	2,816
G07_2	85,877	156	12,339	14,194	78,723	50,981	38,989	12,347	82,722	36,175	17,973	63,159	25	29	3,015	60,301	9,967	2,799
G05_1	82,989	149	11,916	13,677	76,078	49,408	37,823	11,896	79,980	34,706	17,268	61,150	25	27	2,919	59,065	9,432	2,627
G05_2	83,095	147	11,950	13,672	76,295	49,713	38,084	11,864	80,774	34,853	17,107	61,839	28	26	2,880	59,414	9,592	2,715
G02_1	72,597	120	10,287	12,029	66,706	43,458	33,460	10,447	70,895	30,469	14,768	54,603	18	28	2,571	52,178	8,540	2,387
G02_2	72,869	114	10,490	12,133	66,870	43,283	33,066	10,546	70,985	30,784	15,087	53,612	21	24	2,573	51,986	8,575	2,293
G01_1	66,042	116	9,583	11,081	60,624	39,692	30,358	9,643	65,256	28,005	13,135	49,044	20	27	2,382	47,552	7,808	2,023
G01_2	65,707	111	9,442	10,886	60,348	39,576	30,392	9,459	64,596	27,791	13,134	49,590	15	21	2,326	46,653	7,808	2,084

B: iS-VCF

		Number of effects by impact				By functional class			Region									
Data set	Loci	High	Low	Moderate	Modifier	Missense	Nonsense	Synonymous	DOWNSTREAM	EXON	INTERGENIC	INTRON	SPLICE_SITE_ACCEPTOR	SPLICE_SITE_DONOR	SPLICE_SITE_REGION	UPSTREAM	UTR_3_PRIME	UTR_5_PRIME
G07_1	68,458	128	6,033	5,912	64,541	11,534	5,608	5,458	33,937	14,702	49,333	8,101	15	21	552	31,071	2,817	2,077
G07_2	61,139	121	5,869	5,780	57,373	11,312	5,544	5,331	32,435	14,374	42,385	8,039	14	21	545	29,312	2,807	2,063
G05_1	60,237	112	5,659	5,594	56,556	10,831	5,268	5,171	30,992	13,906	42,255	7,626	14	19	523	28,248	2,631	1,967
G05_2	54,080	107	5,519	5,474	50,521	10,638	5,210	5,055	29,735	13,629	36,427	7,568	14	19	516	26,732	2,621	1,955
G02_1	35,517	73	4,025	4,089	32,881	7,512	3,551	3,789	19,685	10,110	22,888	5,233	11	10	344	17,787	1,640	1,423
G02_2	32,284	69	3,935	4,002	29,734	7,385	3,510	3,703	19,025	9,914	19,882	5,192	11	10	341	16,908	1,633	1,415
G01_1	25,163	58	3,206	3,334	23,008	5,979	2,827	3,078	14,754	8,121	15,049	4,214	9	8	280	13,383	1,341	1,204
G01_2	23,097	55	3,142	3,261	21,009	5,892	2,801	3,006	14,287	7,970	13,150	4,187	9	8	277	12,783	1,335	1,203
